# MYC Recruits SPT5 to RNA Polymerase II to Promote Processive Transcription Elongation

**DOI:** 10.1016/j.molcel.2019.02.031

**Published:** 2019-05-16

**Authors:** Apoorva Baluapuri, Julia Hofstetter, Nevenka Dudvarski Stankovic, Theresa Endres, Pranjali Bhandare, Seychelle Monique Vos, Bikash Adhikari, Jessica Denise Schwarz, Ashwin Narain, Markus Vogt, Shuang-Yan Wang, Robert Düster, Lisa Anna Jung, Jens Thorsten Vanselow, Armin Wiegering, Matthias Geyer, Hans Michael Maric, Peter Gallant, Susanne Walz, Andreas Schlosser, Patrick Cramer, Martin Eilers, Elmar Wolf

**Affiliations:** 1 Cancer Systems Biology Group, Theodor Boveri Institute, University of Würzburg, Am Hubland, 97074 Würzburg, Germany; 2 Department of Biochemistry and Molecular Biology, Theodor Boveri Institute, University of Würzburg, Am Hubland, 97074 Würzburg, Germany; 3 Department of Molecular Biology, Max Planck Institute for Biophysical Chemistry, Am Fassberg 11, 37077 Göttingen, Germany; 4 Rudolf Virchow Center for Experimental Biomedicine, Josef-Schneider-Str. 2, 97080 Würzburg, Germany; 5 Institute of Structural Biology, University of Bonn, Sigmund-Freud-Str. 25, 53127 Bonn, Germany; 6 Karolinska Institutet, Department of Biosciences and Nutrition, Hälsovägen 7C, 14157 Huddinge, Sweden; 7 Department of General, Visceral, Transplant, Vascular and Pediatric Surgery, University Hospital Würzburg, Josef-Schneider-Str. 2, 97080 Würzburg, Germany; 8 Core Unit Bioinformatics, Comprehensive Cancer Center Mainfranken, University of Würzburg, Am Hubland, 97074 Würzburg, Germany

**Keywords:** MYC, SPT5, *SUPT5H*, SPT6, RNA polymerase II, transcription, elongation rate, processivity, directionality, tumorigenesis

## Abstract

The MYC oncoprotein binds to promoter-proximal regions of virtually all transcribed genes and enhances RNA polymerase II (Pol II) function, but its precise mode of action is poorly understood. Using mass spectrometry of both MYC and Pol II complexes, we show here that MYC controls the assembly of Pol II with a small set of transcription elongation factors that includes SPT5, a subunit of the elongation factor DSIF. MYC directly binds SPT5, recruits SPT5 to promoters, and enables the CDK7-dependent transfer of SPT5 onto Pol II. Consistent with known functions of SPT5, MYC is required for fast and processive transcription elongation. Intriguingly, the high levels of MYC that are expressed in tumors sequester SPT5 into non-functional complexes, thereby decreasing the expression of growth-suppressive genes. Altogether, these results argue that MYC controls the productive assembly of processive Pol II elongation complexes and provide insight into how oncogenic levels of MYC permit uncontrolled cellular growth.

## Highlights

• MYC enhances productive transcription by defining the protein composition of Pol II
• MYC directly binds SPT5 and hands it over to Pol II in a CDK7-dependent manner
• Transfer of SPT5 increases speed and processivity of Pol II
• MYC’s effects on Pol II function shape its tumor-specific gene expression profile

## Graphical Abstract

**Figure:**
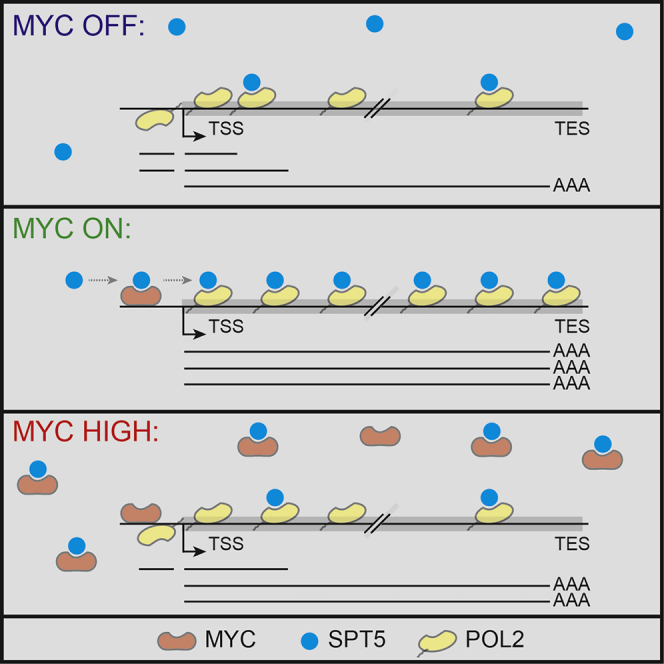

## Teaser

Baluapuri et al. report that MYC governs transition of Pol II into a transcriptionally engaged form by loading the SPT5 protein onto it. This increases Pol II processivity and productive transcription. The effect of MYC on Pol II can regulate expression of those genes, which help tumors to grow.

## Introduction

Deregulated and enhanced expression of the MYC proto-oncogene contributes to the development of most human tumors (Dang, 2012, Schaub et al., 2018), and mouse models demonstrate that many cancer cells depend on high levels of MYC (Gabay et al., 2014). Systemic genetic inhibition of MYC *in vivo* results in rapid regression of established murine tumors, yet it is tolerated by untransformed tissue, defining MYC as a promising therapeutic target (Soucek et al., 2008). A large body of evidence demonstrates that MYC is a nuclear protein that forms a complex with the MYC-associated factor X (MAX) and binds to E-box-containing DNA (CACGTG) (Carroll et al., 2018). The MYC/MAX heterodimer regulates the expression of non-coding transcripts by RNA polymerase I and III, and—most prominently—mRNA expression by RNA polymerase II (Pol II).

Transcription of all coding transcripts starts with the recruitment of Pol II to core promoters. Productive elongation requires the assembly of a highly processive and fast transcriptional apparatus (Jonkers and Lis, 2015). This assembly process entails a series of defined structural transitions of Pol II that are mediated by the differential association of Pol II with auxiliary and regulatory proteins. For example, Pol II transiently pauses transcription after release from the core promoter and continues to elongate upon phosphorylation by the CDK9 kinase (Adelman and Lis, 2012). One crucial protein in this process is the Pol II-associated factor SPT5 (*SUPT5H*), which binds Pol II together with its partner protein, SPT4 (Hartzog et al., 1998, Swanson et al., 1991). In mammals, SPT4 and SPT5 were initially identified as components of a pausing factor named DSIF (DRB sensitivity-inducing factor) (Wada et al., 1998). Recent evidence indicates that SPT5 also travels with Pol II, enhances its processivity (Fitz et al., 2018), and is required for transcriptional elongation (Henriques et al., 2018, Shetty et al., 2017). SPT5 associates with Pol II after the general transcription factor IIE (TFIIE) has dissociated, since the two proteins share the same binding site on Pol II (Grohmann et al., 2011). SPT5 enhances processivity by binding to the DNA exit region on Pol II, facilitating re-winding of upstream DNA and preventing aberrant backtracking of Pol II (Bernecky et al., 2017, Ehara et al., 2017).

Genome-wide chromatin immunoprecipitation sequencing (ChIP-seq) experiments demonstrated MYC binding at the vast majority of open promoters (Lin et al., 2012, Nie et al., 2012, Sabò et al., 2014, Walz et al., 2014). Importantly, MYC promotes an increase in gene expression in lymphoid cells, indicating that MYC’s global binding is productive (Lin et al., 2012, Nie et al., 2012). Moreover, ChIP-sequencing of Pol II demonstrated that MYC globally changes the distribution of Pol II on chromatin (Rahl et al., 2010), and time-resolved analyses indicate that MYC regulates several steps in the transcriptional cycle (de Pretis et al., 2017). Collectively, these data show that MYC exerts a fundamental change in Pol II function.

The precise nature and the molecular mechanisms underlying MYC-mediated changes in Pol II behavior remain elusive. Previous proteomic studies have shown that MYC and the closely related MYCN protein interact with several Pol II-associated protein complexes. Recent systemic interaction studies using BioID indicate that MYC is in proximity to 336 proteins (Kalkat et al., 2018). From these studies, two possible mechanisms to account for MYC’s effects have emerged. First, MYC may recruit CDK9 to promoters to mediate pause release (Rahl et al., 2010). Second, MYC transfers the Pol II-associated factor 1 (PAF1) complex onto Pol II upon ubiquitin-mediated degradation of MYC (Gerlach et al., 2017, Jaenicke et al., 2016). This latter observation raised the possibility that MYC influences the assembly of Pol II with transcription elongation factors, thus changing the behavior of Pol II during productive elongation.

To test this hypothesis, we have combined mass spectrometry experiments of MYC and Pol II with the analyses of nascent transcription as a readout of Pol II behavior to identify rate-limiting steps and mechanistic details in MYC-mediated transcriptional control. Our data show that MYC controls Pol II processivity, directionality, and elongation rate. Mechanistically, we show that MYC recruits SPT5 to core promoters and promotes its transfer onto Pol II in a CDK7-dependent manner. The high levels of MYC found in tumors lead to sequestration of SPT5 and other elongation factors into non-functional complexes, disrupting Pol II assembly and suppressing transcription of a growth-suppressive gene expression program.

## Results

### MYC Controls the Assembly of Transcriptionally Engaged Pol II Complexes

**Figure 1:**
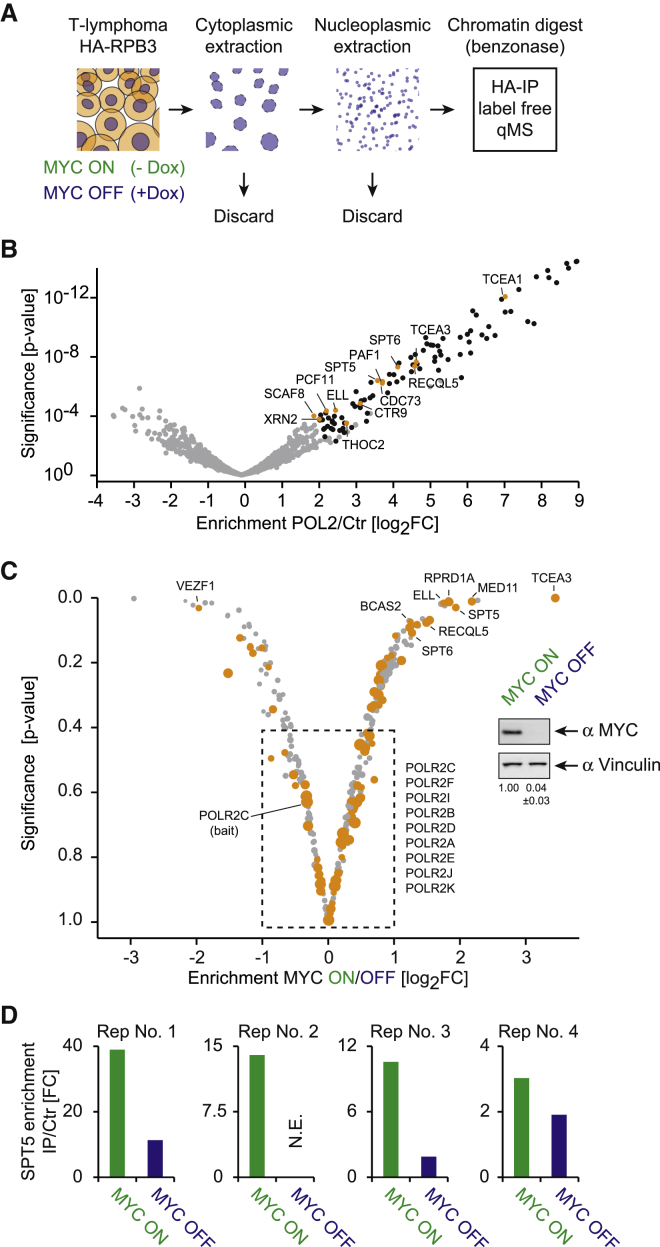
MYC Mediates Changes in Pol II Complex Composition (A) Graphic displaying the method used to identify Pol II-associated proteins. T-lymphoma^MYC-Tet-Off^ cells were stably transfected either with a lentiviral vector expressing HA-tagged RPB3 or an empty lentiviral vector. Cells were harvested, nuclei were isolated, nuclear membranes were lysed, and chromatin-associated proteins were solubilized and used as input for HA-directed immunoprecipitation (IP). Eluted fraction was used in label-free quantitative mass spectrometry (qMS). (B) Volcano plot of the Pol II interactome with transcription elongation factors marked in orange. The x axis displays the enrichment (log_2_FC) of proteins in HA-RPB3-expressing cells compared to control cells (Ctr). The y axis shows the significance (p value) of enrichment calculated from five biological replicate experiments. (C) Volcano plot showing proteins changing their association with Pol II in response to MYC depletion in T-lymphoma^MYC-Tet-Off^ cells. The x axis displays the enrichment of proteins (log_2_FC) between cells expressing (ON) and depleted of (OFF) MYC. Positive values indicate the protein requires MYC to associate with Pol II (e.g., TCEA3 or SPT5). The y axis shows the significance (p value) of enrichment calculated from four biological replicate experiments. Pol II-interacting proteins are shown as orange circles whose size indicates overall enrichment. The insert shows an immunoblot of MYC in T-lymphoma^MYC-Tet-Off^ cells treated with doxycycline (MYC OFF) or ethanol (MYC ON, Vinculin: loading control). (D) Enrichment-values (FC) for SPT5 in the Pol II interactome in the absence and presence of MYC in the four experiments used for the analysis shown in (C). See also Figure S1.

To test whether MYC affects the composition of Pol II complexes, we studied T-lymphoma^MYC-Tet-Off^ cells in which expression of MYC can be suppressed by doxycycline (van Riggelen et al., 2010). To identify Pol II-associated proteins, we adopted a protocol for the native isolation of chromatin-bound Pol II and its associated proteins (Aygün et al., 2008). Cells stably expressing an HA-tagged Pol II subunit (HA-RPB3) were fractionated, and chromatin-bound proteins were solubilized by chromatin digestion. Pol II-associated proteins were isolated by HA-immunoprecipitation (HA-IP) and analyzed by quantitative label-free mass spectrometry (qMS; Figure 1A). Relative to control cells not expressing HA-tagged protein, this analysis identified 101 interacting partners (Table S1) and showed enrichment of components of the integrator and mediator complexes, consistent with previous data (Figure S1) (Aygün et al., 2008). Additionally, we identified numerous transcription-elongation factors as well as splicing and polyadenylation factors (Figure 1B), confirming that we isolated transcriptionally engaged Pol II.

To identify proteins that associate with Pol II in an MYC-dependent manner, we depleted MYC by treating T-lymphoma^MYC-Tet-Off^ cells with doxycycline and compared the Pol II interactome to that of untreated cells (Figure 1C). MYC depletion did not change the abundance of Pol II subunits, indicating that the amount and integrity of Pol II itself was not affected by MYC. One protein, VEZF1, showed a significantly increased association with Pol II upon depletion of MYC (Figure 1C). In contrast, MYC depletion reduced the association of Pol II with the elongation factors RECQL5, SPT6, and SPT5, the splicing factor BCAS2, and the TFIIS subunit TCEA3 (Figures 1C and 1D). These findings suggest that MYC is, directly or indirectly, required for the recruitment of these proteins to chromatin-engaged Pol II complexes.

**Figure 2:**
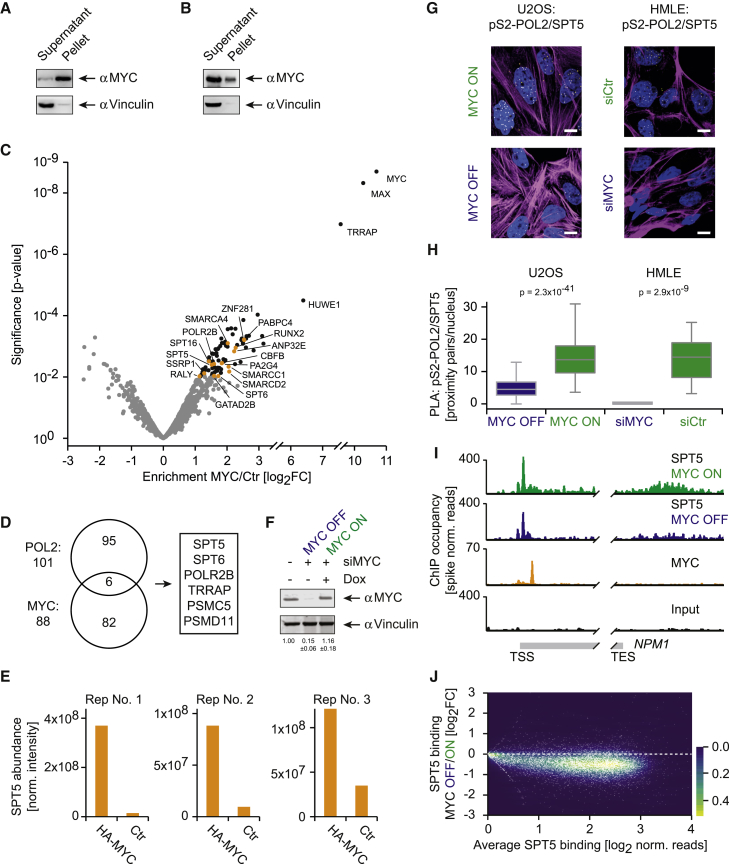
MYC-Dependent Recruitment of SPT5 to Pol II on Chromatin (A and B) Immunoblot showing the solubility of MYC in lysed T-lymphoma^MYC-Tet-Off^ cells in low-salt buffer (A) and in an optimized extraction buffer with benzonase (B) (Vinculin: loading control). (C) Volcano plot of the MYC interactome with uncharacterized interaction partners in orange. The x axis displays the enrichment (log_2_FC) of proteins in HA-MYC-expressing cells compared to control cells. The y axis shows the significance (p value) of enrichment calculated from three biological replicate experiments. (D) Venn diagram depicting the intersection between the Pol II interactome (Figure 1B) and the MYC interactome. (E) Enrichment values (normalized intensity) for SPT5 in the MYC interactome in three experiments (Ctr: control cells not expressing HA-MYC). (F) Immunoblot of MYC in U2OS^MYC-Tet-On^ cells showing endogenous levels, depletion of MYC by siRNA (MYC OFF), and restoration of physiological levels (MYC ON) in cells treated with doxycycline (Dox, Vinculin: loading control). (G) Immunofluorescence images of proximity ligation assays (PLAs) between pS2-Pol II and SPT5 in the absence (MYC OFF, siMYC) and presence (MYC ON, siCtr) of MYC in U2OS^MYC-Tet-On^ and HMLE cells (yellow dots: intensity centers of proximity pairs; blue: Hoechst stained nuclei; magenta: Phalloidin staining; scale bar: 5 μm). (H) Quantitative analysis of PLAs between pS2-Pol II and SPT5 shown in (G). (I) Genome browser pictures of the *NPM1* gene. SPT5 ChIP-RX sequencing experiments were performed in the presence (green) and absence (blue) of MYC (input: black) in U2OS cells. Data for MYC binding (orange) was re-analyzed from a published dataset (Lorenzin et al., 2016). (J) Density plot demonstrating the loss of SPT5 on chromatin in the absence of MYC at single gene level. Each white dot represents a single gene value, which is overlaid on a color gradient indicating the density (SPT5 ChIP-RX sequencing experiments as shown in I). The y axis displays the change of SPT5 binding between the presence and absence of MYC (log_2_FC), and the x axis depicts overall SPT5 binding normalized to gene length (TSS, transcriptional start site; TES, transcriptional end site; norm. normalized). See also Figure S2.

To elucidate which of these factors are recruited to promoters via interaction with MYC, we sought to identify interaction partners of chromatin-associated MYC. Since only a minor fraction of MYC is soluble at low-salt conditions (Figure 2A), we optimized the buffer and included a benzonase treatment step during extraction to liberate most of cellular MYC (Figure 2B). HA-IP followed by qMS identified 88 proteins (Table S2), including well-characterized interaction partners of MYC such as MAX, TRRAP, WDR5, HUWE1, and p400 (Figure S2A), as well as uncharacterized interaction partners (Figure 2C). To identify proteins that are recruited to Pol II directly by MYC, we intersected the sets that interact with MYC and with Pol II and found six common proteins (Figure 2D), but only two changed their association with Pol II upon MYC depletion: SPT5, and, to a lesser degree, SPT6 (Figures 1C and 2E). We confirmed this finding by immunoblotting (Figure S2B).

Next, we examined whether MYC controls the assembly of SPT5 with Pol II in other human cells. First, we transfected U2OS osteosarcoma cells harboring an inducible MYC transgene (U2OS^MYC-Tet-On^) with an siRNA pool targeting MYC, then re-established MYC expression with doxycycline (“MYC ON,” Figure 2F). These cells were compared to cells in which the transgene was not induced after siRNA treatment (“MYC OFF”). As the levels of MYC in doxycycline-treated cells matched endogenous levels (Figure 2F), this experimental design allowed acute manipulation of MYC and minimized the risk of analyzing siRNA-induced off-target effects. Proximity ligation assays (PLAs) between SPT5 and transcriptionally engaged Pol II (pS2-Pol II) showed a clear reduction in nuclear proximity pairs in the absence of MYC (Figures 2G and 2H), even though SPT5 and pS2-Pol II levels remained unchanged (Figures S2C and S2D). These results were not restricted to this cellular system but were also obtained in immortalized human mammary epithelial (HMLE) cells (Figures 2G and 2H).

To determine whether MYC promotes the assembly of SPT5-Pol II complexes on chromatin, we isolated SPT5-bound chromatin by immunoprecipitation (ChIP) from U2OS^MYC-Tet-On^ cells in the absence and presence of MYC and added a defined number of murine cells before precipitation (“spike-in” control; ChIP-RX). ChIP-RX sequencing demonstrated a decrease in SPT5 binding at the promoters and across the entire transcribed region of multiple genes when MYC was depleted (shown for *NPM1* in Figure 2I). Moreover, read distribution at all expressed genes showed a global reduction of SPT5 binding (Figure 2J). Importantly, this effect was not driven by a loss of Pol II association with chromatin, as SPT5 binding was still reduced when normalized to Pol II binding determined by ChIP-RX sequencing of total Pol II (Figures S2E and S2F), indicating that chromatin-associated Pol II binds to less SPT5 in the absence of MYC. Finally, we validated the loss of chromatin-associated SPT5 upon depletion of MYC by ChIP-qPCR (Figure S2G). We concluded that MYC levels are rate-limiting for the association of chromatin-associated Pol II with SPT5.

### MYC Recruits SPT5 by Binding to Its N-Terminal Region

**Figure 3:**
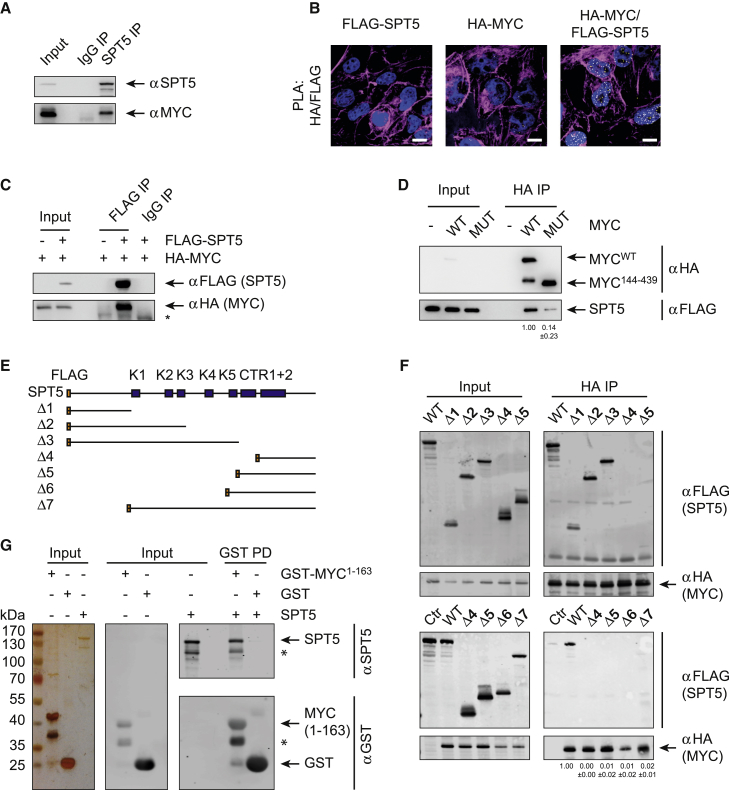
MYC Recruits SPT5 by Binding to its N-terminal Region (A) Immunoblots of endogenous SPT5 IP from HEK293 cells and co-precipitated MYC. Beads coupled to non-specific IgG were used as control. (B) Immunofluorescence images from PLAs. FLAG-SPT5 and HA-MYC were stably co-expressed in U2OS cells by lentiviral transduction, and cells expressing only one protein were used as controls. Proximity between MYC and SPT5 was analyzed with anti-FLAG and anti-HA antibodies (yellow dots: intensity centers of proximity pairs; blue: Hoechst-stained nuclei; magenta: Phalloidin staining; scale bar: 5 μm). (C) Immunoblots of IP experiments. FLAG-SPT5 and HA-MYC were overexpressed by transient transfection in HEK293 cells. FLAG-SPT5 was precipitated and co-precipitating HA-MYC was analyzed by immunoblotting. Cells not expressing FLAG-SPT5 or beads coupled to non-specific IgG were used as controls (^∗^ indicates the antibody heavy chain). (D) Immunoblots of IP experiments. FLAG-SPT5, HA-MYC (WT), and an HA-tagged N-terminal deletion mutant of MYC^144–439^ (MUT) were overexpressed by transient transfection in HEK293 cells. HA-MYC was precipitated and co-precipitating FLAG-SPT5 was analyzed by immunoblotting. Cells not expressing HA-MYC were used as controls. (E) Scheme of FLAG-tagged SPT5 deletion mutants (K: KOW domain; CTR: C-terminal repeat region). (F) Immunoblots of IP experiments. HA-MYC, FLAG-SPT5, and FLAG-tagged deletion mutants of SPT5 shown in (E) were overexpressed by transient transfection in HEK293 cells. HA-MYC was precipitated and co-precipitating FLAG-SPT5 was detected by immunoblotting. Cells not expressing HA-MYC were used as controls (^∗^ indicates signal from IgG chains). (G) Recombinant proteins from pull-down assays visualized by silver staining and immunoblotting. SPT5 (together with SPT4), GST-Myc^1–163^, and GST were isolated from *E. coli* (left, silver staining). GST or GST-Myc^1–163^ were coupled to sepharose and incubated with SPT5. Input and eluted proteins were visualized with antibodies detecting GST or SPT5 (right, immunoblot). See also Figure S3.

First, to better characterize the interaction of MYC with SPT5, we used reciprocal co-immunoprecipitation from HEK293, U2OS, and T-lymphoma cells to confirm interaction of endogenous MYC and SPT5 (Figures 3A and S3A). Consistently, PLAs showed proximity pairs between HA-MYC and FLAG-SPT5 when both proteins were expressed simultaneously but not in control U2OS cells expressing only one of the two proteins (Figure 3B, S3B, and S3C). We also observed efficient co-precipitation when tagged SPT5 and MYC were co-expressed by transient transfection of expression plasmids (Figures 3C and S3D).

To map the domains that mediate this interaction, we expressed a series of deletion mutants of MYC and SPT5 and compared the degree of coimmunoprecipitation (coIP) to that of the full-length proteins. In both proteins, the N-terminal region is critical for their association (Figures 3D–3F). The interaction domain on MYC includes two conserved elements (MYC-box-I/-II) and on SPT5 comprises an unstructured region at the absolute N-terminus and the NGN domain. To test whether the interaction between MYC and SPT5 is direct, we incubated purified SPT5 (Vos et al., 2018) with an N-terminal fragment of MYC (GST-MYC^1–163^), both expressed and isolated from *E. coli*. Efficient co-precipitation of SPT5 was observed with GST-MYC^1–163^ but not with GST alone (Figure 3G). Full-length MYC also interacted with SPT5 upon expression in reticulocyte extracts (Figure S3E) or co-expression in HEK293 cells (Figure S3F). We used this information to test whether the direct interaction between MYC and SPT5 is required for MYC-mediated assembly of Pol II with SPT5 in cells. Therefore, we expressed an HA-tagged mutant of SPT5 lacking the MYC-interaction domain (amino acids 1–271, SPT5–Δ7) and analyzed its proximity to Pol II in comparison to full-length SPT5 by PLAs. Consistent with a positive role of MYC in complex assembly, the proximity between SPT5–Δ7 and Pol II was clearly reduced as compared to full-length SPT5 (Figure S3G). Moreover, depletion of MYC strongly reduced proximity between full-length SPT5 and Pol II but had only small effects on the reduction of proximity between SPT5–Δ7 and Pol II (Figures S3G and S3H). We concluded that the direct interaction of MYC with SPT5 promotes the assembly of SPT5-Pol II complexes.

### CDK7 Activity is Required for Transfer of SPT5 from MYC to Pol II

**Figure 4:**
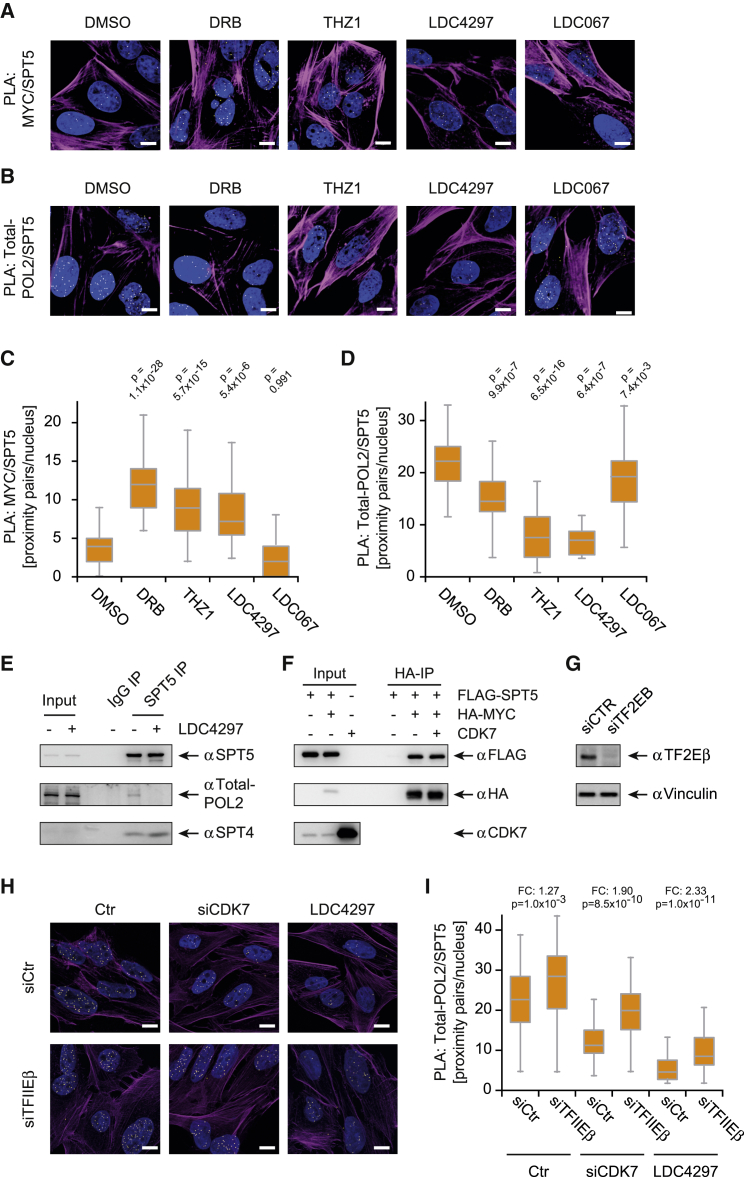
CDK7 Activity Is Required for Transfer of SPT5 from MYC to Pol II (A and B) Immunofluorescence images of PLAs between SPT5 and MYC (A) or SPT5 and total Pol II (B) in cells treated with CDK inhibitors (DRB, THZ1, LDC4297, LDC067) or DMSO vehicle. (C) Quantification of PLAs shown in (A). The number of proximity pairs upon inhibitor treatment was quantified in independent experiments and normalized to the DMSO condition. (D) Quantification of PLAs shown in (B). The number of proximity pairs upon inhibitor treatment was quantified in independent experiments and normalized to the DMSO condition. (E) Immunoblots of endogenous SPT5 precipitated from HEK293 cells treated with LDC4297 or DMSO. Co-precipitated total Pol II and SPT4 were analyzed by immunoblotting. Beads coupled to non-specific IgG were used as controls. (F) Immunoblots of immunoprecipitation experiments. FLAG-SPT5 and HA-MYC were overexpressed by transient transfection in HEK293 cells. HA-MYC was immunoprecipitated and incubated with recombinant CDK7, and co-precipitating FLAG-SPT5 was analyzed by immunoblotting. Cells not expressing HA-MYC were used as control. (G) Immunoblot of TFIIEβ depleted cells. U2OS cells were treated with an siRNA against TFIIEβ or a non-targeting control (Vinculin: loading control). (H and I) Immunofluorescence images (H) and quantification (I) of PLAs between SPT5 and total Pol II. U2OS cells were treated with LDC4297 or an siRNA against CDK7 after depletion of TFIIEβ and in control cells. The number of proximity pairs was quantified and its change in TFIIE-depleted cells to control cells was calculated as fold change (FC). For (A), (B), and (H): yellow dots: intensity centers of proximity pairs; blue: Hoechst stained nuclei; magenta: Phalloidin staining; scale bar: 5 μm. See also Figure S4.

To understand how SPT5 is transferred from MYC onto Pol II, we performed PLAs between MYC, SPT5, and Pol II in U2OS cells pretreated with CDK inhibitors. These experiments used short incubation times at which neither MYC nor SPT5 levels were considerably affected (Figures S4A and S4B). The number of proximity pairs between MYC and SPT5 strongly increased upon treatment with the pan-CDK inhibitor DRB (5,6-dichlorobenzimidazone-1-β-D-ribofuranoside) and the putative CDK7 inhibitors THZ1 (Kwiatkowski et al., 2014) and LDC4297 (Kelso et al., 2014), but not with the CDK9 inhibitor LDC067 (Albert et al., 2014) (Figures 4A and 4C). Conversely, and in agreement with a recent biochemical study (Nilson et al., 2015), proximity between Pol II and SPT5 decreased upon treatment with CDK7 but not CDK9 inhibitors (Figures 4B and 4D). coIP experiments supported a decreased interaction between SPT5 and Pol II upon CDK7 inhibition (Figure 4E). We confirmed a CDK7-specific effect by repeating PLAs in siCDK7-treated cells (Figure S4C–S4E).

Next, we investigated how CDK7 promotes the transfer of SPT5 from MYC onto Pol II. Incubation of MYC-SPT5 complexes with active CDK7 complexes did not affect the interaction of MYC with SPT5 (Figure 4F); therefore, this does not support a model in which MYC or SPT5 are the critical CDK7 substrates. CDK7 promotes the recruitment of mRNA-capping complexes to promoters and reorganizes Pol II complexes by dissociating TFIIE, and both processes may be required for the loading of SPT5 (Larochelle et al., 2012, Nilson et al., 2015, Ohkuma and Roeder, 1994). Depletion of human capping enzyme did not decrease the proximity between SPT5 and Pol II (Figures S4F and S4G), suggesting that MYC-mediated recruitment of SPT5 to Pol II does not depend on mRNA capping. However, depletion of TFIIEβ (Figure 4G) resulted in increased numbers of proximity pairs between SPT5 and Pol II (+27.3%, Figures 4H and 4I), indicating that TFIIE prevents association between SPT5 and Pol II. We hypothesized that the same experiment would show stronger increment of SPT5/Pol II proximity pairs in the absence of CDK7 if CDK7 helps removing TFIIE. Indeed, the increase in SPT5/Pol II proximity upon depletion of TFIIE was even more pronounced when CDK7 was depleted (+133.2%, Figures 4H and 4I) or inhibited (Figures 4H, 4I, S4H, and S4I). These results are compatible with a published model that CDK7 mediates removal of TFIIE from Pol II (Larochelle et al., 2012). Our data indicate that eviction of TFIIE could convert Pol II into a receptive state for SPT5 recruited by MYC but do not exclude the involvement of additional CDK7 targets in this mechanism (Figure S4J).

### MYC Influences Processivity and Directionality of Pol II

**Figure 5:**
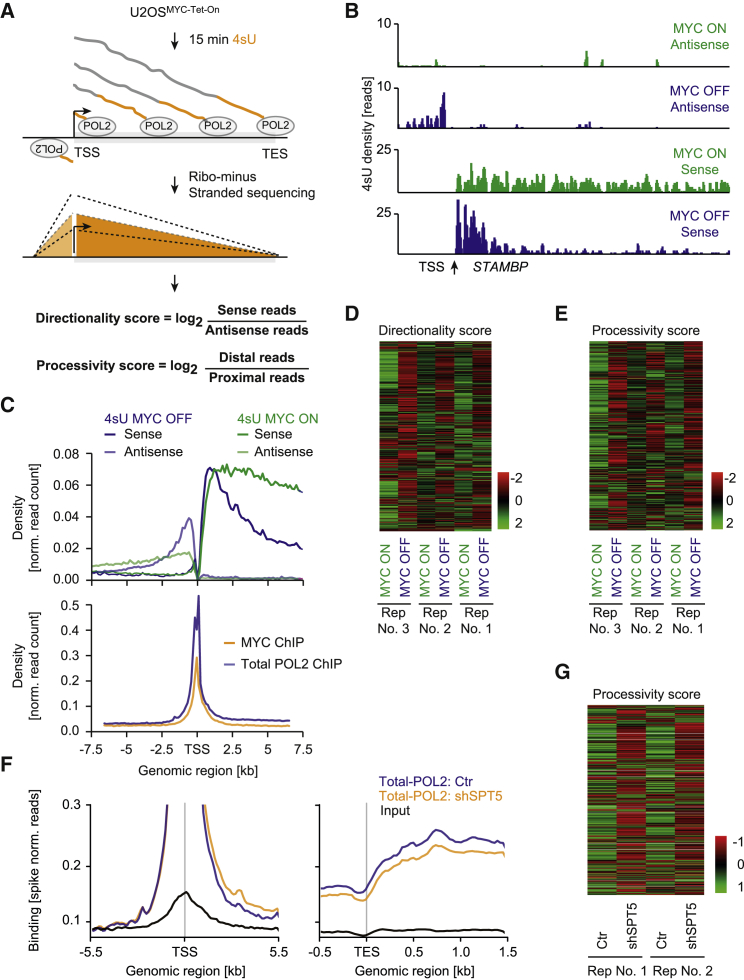
MYC-Mediated Transfer of SPT5 Is Required to Maintain Pol II Processivity (A) Schematic of 4sU sequencing. Nascent transcripts were labeled with 4sU in U2OS^MYC-Tet-On^ cells, converted into strand-specific cDNA and sequenced. Directionality scores were calculated by dividing reads from TSS-TES by TSS-1.5 kb gene regions for all transcribed genes in U2OS cells in the absence and presence of MYC. Processivity scores were calculated by dividing distal (5–7 kb after TSS) by proximal (1-2 kb after TSS) reads. (B) Genome browser pictures of nascent RNA. Example of 4sU signal at the *STAMBP* gene from U2OS cells in the presence and absence of MYC. (C) Average read density of 4sU sequencing experiments (upper panel) in U2OS cells in the absence and presence of MYC. Curves show the spatial distribution of reads independently aligned to sense and antisense strands within 7.5 kb of the TSS for genes longer than 8 kb. Comparison to MYC and Pol II binding in the same region originating from ChIP-sequencing data (lower panel) is shown as average read density (Walz et al., 2014). (D) Heatmap with normalized directionality scores for three replicates calculated in the presence and absence of MYC. Negative values indicate reduced promoter directionality. (E) Heatmap with normalized processivity scores for three replicates calculated in the absence and presence of MYC. Negative values indicate reduced Pol II processivity. (F) Average read density of Pol II ChIP-sequencing experiments around transcriptional start sites (TSS, left) and transcriptional end sites (TES, right). ChIP-RX sequencing was performed with antibodies precipitating total Pol II in U2OS cells depleted of SPT5 by doxycycline-induced shRNA (orange) and control cells (blue; black line: input). (G) Heatmap of normalized processivity scores for two replicates of total Pol II ChIP sequencing in the absence (shSPT5) and presence (Ctr) of SPT5. See also Figure S5.

SPT5 and SPT6 proteins are known to travel with elongating Pol II, and their function was extensively studied in regard to Pol II processivity, transcription elongation rates, and promoter directionality (Ardehali et al., 2009, Henriques et al., 2018). The only protein that increases in the absence of MYC, VEZF1, is associated with Pol II pausing (Gowher et al., 2012). Therefore, we tested whether MYC-mediated changes in Pol II complex composition affect the function of Pol II and analyzed processivity, promoter directionality, and elongation rates. To this end, we labeled newly synthesized transcripts with 4sU (4-thiouridine) (Rabani et al., 2011) and isolated nascent RNAs from cells in the absence and presence of MYC. Then, we prepared strand-specific sequencing libraries to discriminate between transcripts originating from gene transcription and promoter upstream antisense transcription (Figure 5A).

Consistent with multiple previous observations, depletion of MYC decreased RNA synthesis of many genes. Closer inspection of individual genes revealed, however, that the decrease across the transcribed region was not uniform (Figures 5B and S5A). Specifically, the fraction of 4sU-labeled transcripts at distal regions was markedly reduced in the absence of MYC. In contrast, a comparable or even higher number of transcripts of both sense and antisense orientation was detected at regions near the transcriptional start site. An unscaled metagene analysis of all expressed genes (Figures 5C and S5B) confirmed that MYC globally regulates RNA synthesis at distal gene regions. Of note, comparison with Pol II ChIP-sequencing profiles demonstrates that the drop in 4sU signal takes place within the first kilobases of transcription elongation downstream of the Pol II pause site. This indicates that MYC regulates the processivity (number of nucleotide additions per initiation event) of Pol II and that this is a regulatory mechanism after pause release.

To quantify MYC’s effect on Pol II behavior at the level of individual genes, we calculated relative processivity (ratio of distal to proximal 4sU reads) and directionality (ratio of sense to antisense reads) scores for every gene (Core et al., 2012). This analysis revealed that, at most genes, Pol II showed reduced processivity and directionality upon MYC depletion (Figures 5D, 5E, S5D, and S5E). To test whether this function of MYC is conserved for its different isoforms, we repeated the 4sU sequencing experiment in SH-EP neuroblastoma cells that express a MYCN-estrogen receptor chimeric protein. Activation of MYCN increased the processivity of transcription (Figure S5C). We concluded that MYC and MYCN increase the processivity of Pol II and that MYC proteins are required for the transcription of distal regions of long genes.

To test whether MYC’s effects on Pol II elongation are mediated by the loading of SPT5, we depleted SPT5 by acute induction of a stably integrated, doxycycline-inducible shRNA (Figure S5F) and performed Pol II ChIP-RX and 4sU sequencing experiments. ChIP signals of both total Pol II (Figure 5F) and pS2-Pol II (Figure S5G) in SPT5-depleted cells were higher immediately downstream of the transcriptional start site but successively decreased towards the 3′ end of gene bodies as compared to control cells. Consequently, we observed that the processivity score is reduced at most genes (Figure 5G) and 4sU sequencing confirmed reduced processivity upon depletion of SPT5 (Figure S5H). We concluded that MYC affects transcriptional elongation downstream of the transcriptional start site by promoting the assembly of SPT5 and Pol II. However, these results do not exclude the possibility of involvement of other mechanisms of transcriptional regulation. Upon SPT5 depletion, we did not observe a re-distribution of Pol II molecules between the sense and antisense regions of Pol II-transcribed genes, indicating that MYC’s effect on promoter directionality is mediated by an additional factor.

### MYC Maintains High Pol II Elongation Rates

**Figure 6:**
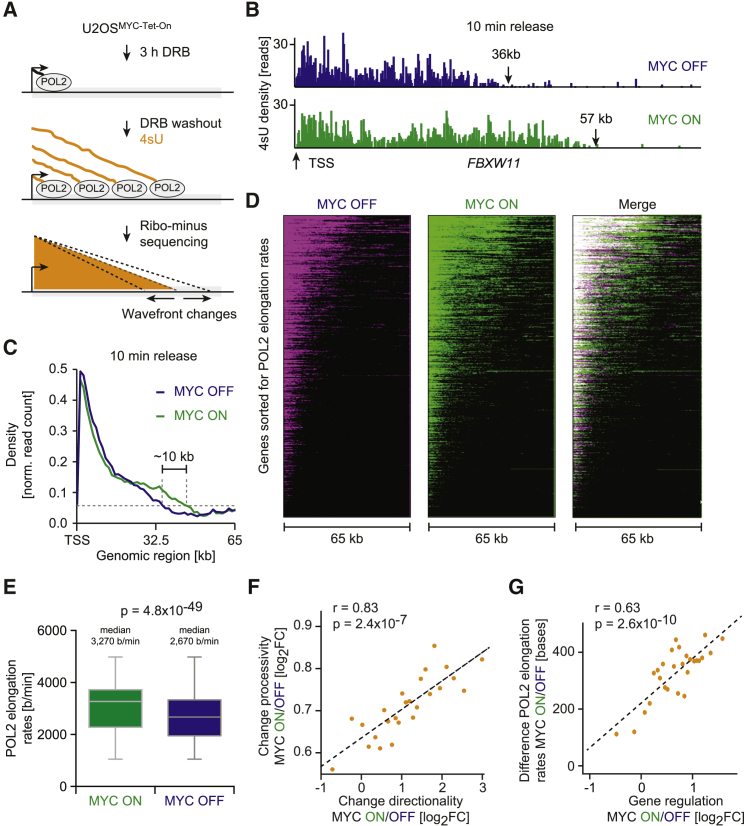
MYC Is Required to Globally Maintain High Transcription Elongation Rates (A) Schematic of 4sU-DRB sequencing. U2OS^MYC-Tet-On^ cells were treated with DRB to inhibit Pol II molecules from starting transcriptional elongation. DRB was washed out, and nascent transcripts were labeled with 4sU, isolated, converted into cDNA, and sequenced. (B) Genome browser pictures of the *FBXW11* gene in a 4sU-DRB sequencing experiment. The wave front indicates the location of Pol II 10 min after release from DRB inhibition in the presence (57 kb) and absence (36 kb) of MYC. (C) Density profile of 4sU-DRB sequencing reads for 3,732 genes (50–100 kb long) 10 min after DRB release. The reads were aligned to each TSS and averaged. (D) Heatmaps of normalized 4sU reads (10 min after DRB release) in the absence and presence of MYC sorted for elongation rates. (E) Pol II elongation rates in the presence and absence of MYC measured at a 10 min release time point. (F) Scatterplot analyzing the correlation between MYC-mediated changes in Pol II directionality and processivity. Mean values of bins containing an equal number of genes are shown. The x axis displays the change in promoter directionality by comparing the directionality score (log_2_FC) in the presence and absence of MYC (MYC ON, MYC OFF) based on 4sU sequencing experiments. The y axis depicts the change in the processivity score. (G) Scatterplot of the correlation between MYC-mediated changes in Pol II elongation rates (y axis) and in gene regulation (x axis) in 4sU-DRB-sequencing experiments. Mean values of bins containing an equal number of genes are shown (r: correlation coefficient). See also Figure S6.

Since low transcriptional processivity and directionality has been associated with low Pol II elongation rates (Fong et al., 2017, Mason and Struhl, 2005), we determined whether MYC influences Pol II elongation rates (nucleotides per minute). Pol II elongation rates in living cells can be measured by 4sU-DRB sequencing (Fuchs et al., 2014), which involves the analysis of nascent transcription after the reversible inhibition of transcription by DRB (Figure 6A). In contrast to Pol II ChIP sequencing, this technique allows discrimination between effects on transcriptional elongation and those on initiation and pause release.

First, as Pol II elongation rates vary between different cell types, we measured elongation rates in U2OS cells by allowing transcription to proceed in the presence of 4sU for 4, 8, or 16 min after DRB washout. As expected, inspection of individual genes showed that the length of nascent RNAs increased over time, and the position of ceasing 4sU-signal (“transcriptional wave front”) indicated the distance traveled by the first Pol II molecules after DRB washout (Figure S6A). Elongation rates varied among genes (2.4–3.2 kb/min; Figure S6B), in agreement with previous reports (Danko et al., 2013, Saponaro et al., 2014).

To analyze the effect of MYC on Pol II elongation rates, we performed a 4sU-DRB sequencing experiment at two different release times after DRB treatment. In MYC-depleted cells, nascent RNA density close to the transcriptional start site was unaltered compared to cells expressing MYC (Figures 6B and S6C). In contrast, the transcriptional wave front of MYC-depleted cells was significantly behind that of MYC-expressing cells (e.g., 36 versus 57 kb for *FBXW11*; Figure 6B). Analysis of the averaged nascent RNA signal at all long genes showed a recession of the transcriptional wave front by about 10 kb in the absence of MYC (Figure 6C). Importantly, the decrease in Pol II elongation rate was not driven by a small fraction of highly expressed genes, as heatmap analysis demonstrated a global effect (Figure 6D). To measure MYC’s effect on elongation at the gene level, we calculated elongation rates for every gene and found that the median elongation rate decreased significantly from 3,270 bases/min in the presence of MYC to 2,670 bases/min in its absence (Figures 6E and S6D). We concluded that MYC is required for maintaining high-Pol II elongation rates and wondered whether MYC’s effects on elongation account for MYC-dependent changes in gene expression.

In agreement with studies analyzing slow Pol II mutants (Fong et al., 2017), we observed that the effects of MYC on elongation, promoter directionality, and Pol II processivity correlated with each other at a gene level (Figure 6F). More importantly, not only did we find that MYC-dependent changes in elongation rate correlated with MYC-mediated gene regulation (Figure 6G) but also discovered that the change in promoter directionality stratifies gene regulation better than changes in initiation or traveling ratio (Figure S6E). Further, we ranked all genes for MYC’s effect on elongation and performed gene set enrichment analyses (GSEA). This analysis indicated that genes with higher elongation rates and increased promoter directionality in the presence of MYC were enriched in well-known sets of MYC-induced genes (Figures S6F and S6G). Mechanistically, changes in Pol II elongation can influence gene expression by changes in the degree of premature termination. Intriguingly, the drop in processivity in the absence of MYC was most apparent at the first alternative polyadenylation site (Figure S6H). We concluded that the MYC-dependent effects on transcriptional elongation establish MYC-mediated gene expression profiles by increasing Pol II processivity and suppressing premature termination.

### MYC’s Effects on Pol II Function Shape Its Tumor-Specific Gene Expression Profile

**Figure 7:**
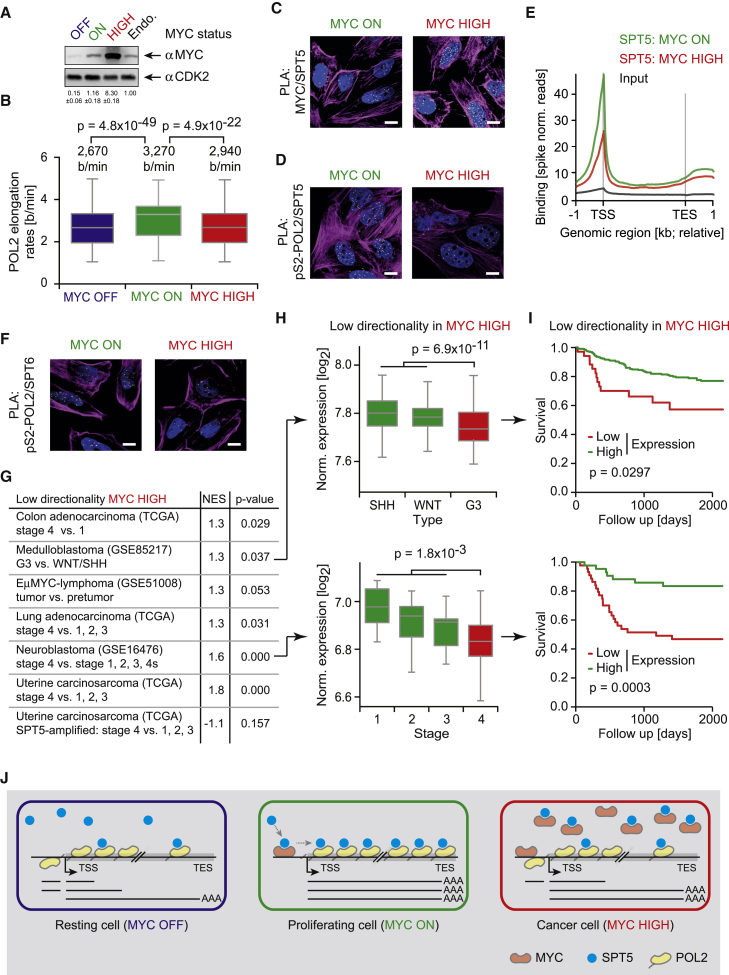
Oncogenic MYC Levels Sequester SPT5 away from Pol II (A) Immunoblot of U2OS^MYC-Tet-On^ cells depleted of MYC (MYC OFF: siMYC), in the “MYC ON” condition (siMYC, doxycycline) with oncogenic levels (MYC-HIGH: doxycycline) and untreated to show endogenous levels (Endo.). (B) Pol II elongation rates in the absence (OFF) and presence of MYC (ON) and at oncogenic MYC levels (HIGH). (C and D) Immunofluorescence images of PLAs between MYC and SPT5 (C) and pS2-Pol II and SPT5 (D) in U2OS^MYC-Tet-On^ cells at normal and oncogenic levels of MYC. (E) Metagene analysis of SPT5 ChIP-RX-sequencing experiments in U2OS^MYC-Tet-On^ cells at normal (green) and oncogenic (red) levels of MYC (Input: black; norm., normalized; TSS, transcriptional start site; TES, transcriptional end site). (F) Immunofluorescence images of PLAs between pS2-Pol II and SPT6 in U2OS^MYC-Tet-On^ cells at normal and oncogenic levels of MYC. (G) Gene set enrichment analyses of gene-expression profiles from different types of tumors using a set of genes with low directionality scores in high MYC conditions (n = 300) and comparing high- and low-grade tumors. (NES, normalized enrichment score). A positive NES value indicates activation of the respective gene set in low-grade tumors. (H) Normalized gene expression of 300 genes with low directionality scores in high MYC in tumors of different medulloblastoma types (top) and neuroblastoma stages (bottom). (I) Kaplan-Meier survival curves for patients with medulloblastoma (top) and neuroblastoma (bottom), stratified by the expression of 300 genes with the lowest directionality score in high MYC. (J) Model of MYC function during transcriptional elongation: in growing cells (middle), MYC binds SPT5, recruits it to promoters and transfers it to the transcriptional machinery before transcription elongation. As a consequence, SPT5-loaded Pol II produces full-length transcripts via fast, processive, and directional transcription. In resting cells (left), SPT5 is insufficiently recruited and Pol II loses directionality and processivity, resulting in an increase of antisense and abortive transcripts. In cancer cells with high MYC levels (right), a significant fraction of SPT5 is sequestered by soluble MYC and, as a consequence, transcription is reduced at genes that belong to known MYC-repressed genes. For (C), (D), and (F): yellow dots: intensity centers of proximity pairs; blue: Hoechst stained nuclei; magenta: Phalloidin staining; scale bar: 5 μm. See also Figure S7.

Like many normal cells, U2OS cells express about 10^5^ MYC molecules. In contrast, most tumor cells express higher MYC levels, with multiple myeloma and colorectal cancers expressing up to 10^6^ MYC molecules per cell (Lin et al., 2012, Lorenzin et al., 2016). The “MYC ON” situation studied so far corresponds to endogenous MYC levels in U2OS^MYC-Tet-On^ cells. To study the effects of oncogenic MYC levels on transcriptional elongation, we induced high MYC expression by treating U2OS^MYC-Tet-On^ cells with doxycycline without previous siRNA-mediated MYC silencing (Figure 7A). Doxycycline-induced cells express approximately 3^∗^10^6^ MYC molecules per cell and display tumor-specific gene expression patterns (Figure S7A) (Lorenzin et al., 2016). Surprisingly, high MYC levels had a significant inhibitory effect on Pol II elongation rates compared to cells with endogenous MYC levels (Figure 7B). We wondered whether oncogenic levels of MYC sequester SPT5 away from Pol II, a phenomenon termed squelching. Indeed, PLAs of U2OS cells expressing high MYC levels showed increased proximity between MYC and SPT5 (Figures 7C and S7B) but decreased proximity between SPT5 and Pol II (Figures 7D and S7C). Similar results were seen in HeLa cells (Figures S7D and S7E). Moreover, ChIP-RX sequencing showed reduced chromatin association of SPT5 at high MYC levels (Figure 7E). In addition to SPT5, high MYC levels also induce squelching of SPT6 as measured by PLAs between SPT6 and Pol II (Figures 7F and S7F).

We wondered whether MYC-mediated squelching of SPT5 and SPT6 influences transcriptional elongation. 4sU sequencing experiments demonstrated that certain genes lose processivity and directionality at oncogenic levels of MYC (Figures S7G and S7H). Intriguingly, GSEA using the decrease in directionality as a stratifying parameter established that this decrease was strongest for sets of genes that are well established as MYC-repressed genes (Figure S7I), including immune response genes (Figure S7J) and genes encoding components of the TGFβ pathway (Figure S7K).

Previously, we showed that the gene expression patterns established at oncogenic levels of MYC in cultured cells are found in many advanced tumors and that gene repression significantly contributes to this (Walz et al., 2014). Intriguingly, the decrease in directionality induced by oncogenic MYC levels was a powerful stratification for tumor stage in well-characterized MYC-driven tumor entities (Figure 7G). For instance, genes that lose directionality at high MYC levels were expressed at reduced levels in MYC-driven group 3 medulloblastoma and aggressive neuroblastoma compared to less aggressive sub-entities (Figure 7H). Accordingly, patients with the lowest expression of these genes had a worse prognosis (Figure 7I). Intriguingly, this gene signature was also repressed in the majority of aggressive sub-entities of uterine carcinoma but not in tumors with amplification of the *SUPT5H* gene, which encodes SPT5 (Figure 7G). This finding re-enforces the notion that MYC-mediated repression involves squelching of SPT5. We concluded that transcriptional repression at high MYC levels involves a decrease in transcription elongation that contributes to the establishment of tumor-specific gene expression signatures.

## Discussion

Upon recruitment to promoters, Pol II undergoes a series of transitions during which it progressively associates with transcription elongation and RNA processing factors to form an elongation-competent complex. Previous work has shown that MYC plays a central role in this assembly process, but the precise function had not been clarified. Here, we show that MYC induces a major transition in Pol II action from a slow and poorly processive to a fast, directional, and highly processive mode (Figure 7J).

To understand the underlying molecular mechanisms, we have analyzed the Pol II interactome at different MYC levels and identified proteins that associate with Pol II in a MYC-dependent manner. We found that levels of MYC are rate limiting for the association of Pol II with SPT5 and SPT6. Our data demonstrate that MYC directly binds to SPT5, recruits it to promoters, and facilitates the CDK7-dependent assembly of Pol II-SPT5 complexes (Figure S4J). Importantly, MYC-dependent changes in processivity and directionality, rather than effects on initiation and pause release, parallel MYC’s effects on steady-state mRNA levels.

Depletion of SPT5 and the comparison of our data with known properties of SPT5 (Fitz et al., 2018, Henriques et al., 2018, Shetty et al., 2017) show that the MYC-dependent increase in processivity can be accounted for by the transfer of SPT5, while the effect on directionality is independent of SPT5 and may reflect the MYC-dependent transfer of other elongation factors, such as the PAF1 complex, onto Pol II. It was also suggested that MYC binds to CDK9 (the catalytical subunit of pTEFb) (Eberhardy and Farnham, 2001) and recruits it to promoters (Rahl et al., 2010). This is relevant, as SPT5 is a crucial target of CDK9. Therefore, we speculate that MYC, by promoting the assembly of Pol II with SPT5, generates a substrate for pTEFb and subsequently promotes conversion of SPT5 from a pause factor to a positive elongation factor. The notion that MYC activates transcription by more than one mechanism is in agreement with experimental observations and theoretical considerations that efficient transcription activation requires the reduction of several energy barriers within the activation cycle of Pol II transcription (Kouzine et al., 2013, Michel and Cramer, 2013). Future analyses on the structural nature of the interaction between MYC and SPT5, CDK9, and the PAF1 complex will allow the design of interaction-defective mutants and will give further insights about the precise role of each interaction in MYC-mediated transcriptional activation.

Why does MYC regulate Pol II processivity and directionality in metazoans? Recent work has shown that the process of transcription establishes specific chromatin modifications (Fong et al., 2017), which prevent heterochromatinization and stable silencing of active promoters. One intriguing possibility emerging from these observations is that a major function of transcription in quiescent cells is to maintain promoters in an open chromatin structure that is responsive to external stimuli such as growth factors and nutrients. At the same time, the instability of abortive transcripts generated in quiescent cells would enable the rapid re-cycling of nucleotides and prevent depletion of cellular nucleotide pools. Growth-stimuli-induced MYC activation would then rapidly switch transcription to a productive mode to generate stable mRNAs and build up biomass. Comparing quiescent to growing cells, our data are compatible with models postulating a global function of MYC in the generation of functional coding transcripts to support growth in response to mitogenic stimulation (Lin et al., 2012, Nie et al., 2012).

Previous work has also established that gene expression profiles of MYC-driven human tumors are shaped by both transcriptional activation and repression of specific genes (Muhar et al., 2018, Sabò et al., 2014, Walz et al., 2014), and a major question for the field has been how and why a global transcriptional activator generates specific gene expression profiles (Kress et al., 2015, Wolf et al., 2014). We and others have shown previously that differences in MYC’s promoter affinity are a major factor shaping expression (de Pretis et al., 2017, Lorenzin et al., 2016). The data presented here argue that specificity of regulation is largely generated by the mechanism by which MYC regulates Pol II function. Specifically, we observed that the high levels of MYC that are expressed in human tumors do not further enhance transcriptional elongation but rather induce squelching of SPT5, reducing the processivity of Pol II-dependent transcription (Figure 7J). Conversely, it is unlikely that a further increase in global transcription rate accounts for the selective pressure to increase MYC levels during tumor progression. Rather, sequestration of SPT5 by oncogenic levels of MYC decreases Pol II processivity and directionality on genes that are known targets of MYC-dependent repression, for example genes encoding proteins of the TGFβ pathway and regulators of the interactions of tumor cells with the immune system. Therefore, one possibility is that tumors exploit MYC-dependent sequestration of SPT5 to repress tumor-suppressive genes, providing an explanation for the selection of high levels of MYC during tumor progression.

It is noteworthy that amplification of the *SUPT5H* gene (encoding SPT5) is prevalent in uterine, ovarian, and pancreatic carcinoma (>10% of cases) (Gao et al., 2013). Our analysis of gene expression profiles of uterine carcinomas with *SUPT5H* amplifications suggested that these amplifications enable such tumors to escape MYC-dependent transcriptional repression. Oncogenic levels of MYC are stressful to cells and sensitize them to many pro-apoptotic stimuli (Murphy et al., 2008). Intriguingly, this sensitization has been linked both to MYC-dependent transcriptional repression and to global stalling of Pol II in nutrient-deprived situations (Dejure et al., 2017). Therefore, we propose that the amplification of *SUPT5H* enables tumor cells to escape MYC-driven apoptosis. Conversely, the mechanisms that alter the composition of the elongation complex also contribute to the adaptations to selective pressures exerted by oncogenic levels of MYC. These mechanisms may constitute specific vulnerabilities of MYC-driven tumor cells; hence they may be the basis of new forms of therapy.

## STAR★Methods

### Key Resources Table

**Table:** 

REAGENT or RESOURCE	SOURCE	IDENTIFIER
**Antibodies**		

Rabbit monoclonal anti-MYC (Y69)	Abcam	Cat#ab32072; RRID: AB_731658; Lot: GR295111-2/33/45
Mouse Monoclonal anti-MYC (C33)	Santa Cruz Biotechnology	Cat#sc-42 RRID: AB_2282408 Lot: L1813
Mouse monoclonal anti-vinculin, clone^∗^hv	Sigma	Cat#V9131; RRID: AB_477629; Lot: 034M4809V
Rabbit polyclonal anti-HA-probe (Y-11) X	Santa Cruz Biotechnology	Cat#sc-805X; RRID: AB_631618; Lot: H1215
Rabbit monoclonal anti-SUPT5H [EPR5145(2)]	Abcam	Cat#ab126592; RRID: AB_11128976; Lot: GR79342-8 + GR155828-1
Mouse monoclonal anti-SPT5 (D-3) X	Santa Cruz Biotechnology	Cat#sc-133217X; RRID: AB_2196394; Lot: B1309
Rabbit polyclonal anti-SPT6	Novus Biologicals	Cat#NB100-2582; RRID: AB_609125; Lot: A1
Mouse monoclonal anti-POLR2B (E-12)	Santa Cruz Biotechnology	Cat#sc-166803; RRID: AB_2167499; Lot: G2117
Rabbit polyclonal anti-TRRAP Antibody AbVantage™ Pack	Bethyl Laboratories	Cat#A310-373A; RRID: AB_873156
Rabbit polyclonal anti-RNA polymerase II CTD repeat YSPTSPS (phospho S2) antibody - ChIP Grade	Abcam	Cat#ab5095; RRID: AB_304749; Lot: GR3205578-1
Mouse anti-RNA Polymerase II RPB1 Antibody (H5 Clone) (phospho S2) antibody	BioLegend	Cat#920203 RRID: AB_2734687 Lot:B223109
Rabbit monoclonal anti-CDK2 (78B2)	Cell Signaling Technology	Cat#2546S; Lot: 6
Mouse monoclonal Anti-FLAG M2	Sigma	Cat#F3165; RRID: AB_259529; Lot: SLBQ7119V + SLBN8915V
Goat polyclonal anti-GST	Sigma	Cat#GE27-4577-01; Lot: 362611
Mouse monoclonal anti-Pol II (A-10)	Santa Cruz Biotechnology	Cat#sc-17798; RRID: AB_677355; Lot:B0717
Mouse monoclonal anti-Pol II (F-12)	Santa Cruz Biotechnology	Cat#sc-55492 RRID: AB_630203 Lot: F2416
Mouse monoclonal anti-SPT4 (A-12) X	Santa Cruz Biotechnology	Cat#sc-515238X; Lot: H0316 + I2717
Mouse monoclonal anti-CDK7 (MO-1)	Santa Cruz Biotechnology	Cat#sc-56284; RRID: AB_1121427; Lot: J2108
Mouse Monoclonal anti-TF2EB (A-1)	Santa Cruz Biotechnology	Cat#sc-137000 RRID: AB_2114531 Lot: F0816
ECL-Anti-rabbit IgG Horseradish Peroxidase	GE Healthcare / Fisher Scientific GmbH	Cat#1079-4347 / GEHENA934
ECL-Anti-mouse IgG Horseradish Peroxidase	GE Healthcare / Fisher Scientific GmbH	Cat#1019-6124 / GEHENA931
Donkey polyclonal anti-goat IgG-HRP secondary antibody	Santa Cruz Biotechnology	Cat#sc-2020
IRDye® 800CW Donkey anti-Rabbit IgG (H + L)	LI-COR Biosciences GmbH	Cat#926-32213
IRDye® 680RD Donkey anti-Mouse IgG (H + L)	LI-COR Biosciences GmbH	Cat#926-68072
Goat anti-Rabbit IgG (H+L) Highly Cross-Adsorbed Secondary Antibody, Alexa Fluor 568	Thermo Fisher Scientific	Cat#A-11036
Goat anti-Mouse IgG (H+L) Highly Cross-Adsorbed Secondary Antibody, Alexa Fluor 488	Thermo Fisher Scientific	Cat#A-11029

**Bacterial and Virus Strains**		

pRRL-puro	Walz et al., 2014	N/A
pRRL-puro-RPB3-HA	This paper	N/A
pRRL-puro-MYC-WT-HA	This paper	N/A
pRRL-puro-MYC^144–439^-HA	This paper	N/A
pRRL-puro-N-HA-SPT5	This paper	N/A
pRRL-N-HA-SPT5_Δ7	This paper	N/A
pIND11	Fellmann et al., 2013	N/A
pIND11-h-SPT5-mirE1	This paper	N/A
pLT3	Fellmann et al., 2013	N/A
pLT3-h-SPT5-mirE3	This paper	N/A

**Chemicals, Peptides, and Recombinant Proteins**		

Benzonase nuclease purity >99% 25U/μl	Merck Millipore	Cat#70664-3
Puromycin	InvivoGen	Cat#ant-pr-1
Doxycyclin	Sigma	Cat# D9891
Protease Inhibitor Cocktail	Sigma	Cat#P8340
Phosphatase Inhibitor Cocktail 2	Sigma	Cat#P5726
Phosphatase Inhibitor Cocktail 3	Sigma	Cat#P0044
Hoechst 33342	Thermo Fisher Scientific	Cat#62249
Alexa 488-conjugated phalloidin	Thermo Fisher Scientific	Cat#A12379
DRB (5,6-Dichlorobenzimidazole 1-β-D-ribofuranoside)	Sigma	Cat#D1916
THZ1 Hydrochloride	MedChem Express / Hycultec GmbH	Cat#HY-80013A
LDC000067	Selleckchem / Biozol	Cat#SEL-S7461
LDC4297	Selleckchem / Biozol	Cat#SEL-S7992
4-thiouridine (4sU)	Sigma	Cat#T4509
L-Cystein, L-Methionin S35-label	Hartmann Analytic	Cat#SCIS103/37
Actinomycin D	Sigma	Cat# A9415
Pierc Anti-HA Magnetic Beads	Thermo Fisher Scientific	Cat#88836
Dynabead Protein A for Immunoprecipitation	Thermo Fisher Scientific	Cat#10002D
Dynabead Protein G for Immunoprecipitation	Thermo Fisher Scientific	Cat#10004D
NuPAGE LDS Sample Buffer (4X)	Thermo Fisher Scientific	Cat#NP0007
DSIF	Vos et al., 2018	N/A
CDK7	Boehning et al., 2018	N/A
GST	This paper	N/A
GST-MYC^1–163^	This paper	N/A

**Critical Commercial Assays**		

PLA Duolink kits	Sigma	Cat#DUO92008, Cat#DUO92004, Cat#DUO92002
TNT® T7 Quick Coupled Transcription/Translation System	Promega	Cat#L1171
Quant-iT PicoGreen dsDNA assay	Thermo Fischer Scientific	Cat#P7589
Quant-iT RiboGreen RNA Assay Kit	Thermo Fischer Scientific	Cat#R11490
NEBNext® ChIP-Seq Library Prep Master Mix Set for Illumina®	NEB	Cat#E6240 S
NEBNext® Ultra™ Directional RNA Library Prep Kit for Illumina	NEB	Cat#E7420 S
NEBNext® rRNA Depletion Kit (Human/Mouse/Rat)	NEB	Cat#E6310
RNeasy MinElute Cleanup Kit	QIAGEN	Cat#74204
miRNeasy MiniKit	QIAGEN	Cat# 217004
Dynabeads® MyOne™ Streptavidin T1	Thermo Fischer Scientific	Cat#65601
Pierce™ DTT (Dithiothreitol), No-Weigh™ Format	Thermo Fischer Scientific	Cat# 20291
NextSeq® 500/550 High Output Kit v2 (75 cycles)	Illumina	Cat#FC-404-2005
ON-TARGETplus Human MYC SMARTpool	Horizon Discovery Group	Cat#L-003282-02-0050
ON-TARGETplus Non-targeting Pool	Horizon Discovery Group	Cat#D-001810-10-50
ON-TARGETplus Human GTF2E1 SMARTpool	Horizon Discovery Group	Cat#L-006531-00-0005
ON-TARGETplus Human GTF2E2 SMARTpool	Horizon Discovery Group	Cat#L-017725-00-0005
ON-TARGETplus Human CDK7 SMARTpool	Horizon Discovery Group	Cat#L-003241-00-0005
ON-TARGETplus Human SPT5 SMARTpool	Horizon Discovery Group	Cat#L-016234-00-0020
ON-TARGETplus Human HCE1 SMARTpool	Horizon Discovery Group	Cat#L-009782-00-0005

**Deposited Data**		

Raw and analyzed data	This paper	GEO: GSE115365
Raw and analyzed data	This paper	GEO: GSE113861
Raw and analyzed data	Walz et al., 2014	GEO: GSE44672
Raw and analyzed data	Lorenzin et al., 2016	GEO: GSE77356
Human reference genome GRCh37/hg19	Genome Reference Consortium	https://support.illumina.com/sequencing/sequencing_software/igenome.html
Mouse reference genome mm10	Genome Reference Consortium	https://support.illumina.com/sequencing/sequencing_software/igenome.html

**Experimental Models: Cell Lines**		

U2OS	ATCC	N/A
U2OS^MYC-Tet-On^	Walz et al., 2014	N/A
T-lymphoma^MYC-Tet-Off^	van Riggelen et al., 2010	N/A
HEK293	ATCC	N/A
HeLa	ATCC	N/A
HMLE	Weinberg Lab	N/A
NIH 3T3	ATCC	N/A
SH-EP MYCN-ER	Eilers Lab	N/A

**Oligonucleotides**		

Primer for cloning: Table S3	This paper	N/A
shSPT5 human mirE1: TGCTGTTGACAGTGAGCGCAAGAAGCTGTTTGG TCTAAAATAGTGAAGCCACAGATGTATTTTAGAC CAAACAGCTTCTTTTGCCTACTGCCTCGGA	This paper	N/A
shSPT5 human mirE3: TGCTGTTGACAGTGAGCGAAGGGACCAGCGAG AAGAAGAATAGTGAAGCCACAGATGTATTCTTC TTCTCGCTGGTCCCTCTGCCTACTGCCTCGGA	This paper	N/A
Primer qPCR NCL_f: TACTGGGCAGGCTCAGTCTT	This paper	N/A
Primer qPCR NCL_r: GAAGATCCCGGAGCACGTA	This paper	N/A
Primer qPCR NegReg_f: TTTTCTCACATTGCCCCTGT	This paper	N/A
Primer qPCR NegReg_r: TCAATGCTGTACCAGGCAAA	This paper	N/A

**Recombinant DNA**		

psPAX2	Trono Laboratory	Addgene 12260
pMD2.G	Trono Laboratory	Addgene 12259
pCMV3-N-FLAG-SPT5	ABclonal	Cat#HG18941-NF
pCMV3-N-FLAG-SPT5_Δ1	This paper	N/A
pCMV3-N-FLAG-SPT5_Δ2	This paper	N/A
pCMV3-N-FLAG-SPT5_Δ3	This paper	N/A
pCMV3-N-FLAG-SPT5_Δ4	This paper	N/A
pCMV3-N-FLAG-SPT5_Δ5	This paper	N/A
pCMV3-N-FLAG-SPT5_Δ6	This paper	N/A
pCMV3-N-FLAG-SPT5_Δ7	This paper	N/A
pCDNA3	Invitrogen	N/A
pCDNA3-MYC-WT	Vo et al., 2016	N/A
pGex-4T3	Pharmacia	N/A
pGex-4T3-MYC^1–163^	This paper	N/A

**Software and Algorithms**		

Max Quant (version 1.5.3.12) performed with Andromeda	Cox and Mann, 2008	http://www.coxdocs.org/doku.php?id=:maxquant:start
ImageJ (version 1.50h)	Schneider et al., 2012	https://imagej.net/ImageJ
Image Studio Lite (Version 5.2.5)	LI-COR Biosciences - GmbH	https://www.licor.com/bio/products/software/image_studio_lite/
Bowtie v1.2	Langmead et al., 2009	http://bowtie-bio.sourceforge.net/index.shtml
Bowtie v2.3.2	Langmead et al., 2009	http://bowtie-bio.sourceforge.net/index.shtml
Bedtools v2.26.0	Quinlan, 2014	https://github.com/arq5x/bedtools2/releases
SAMtools v1.3	N/A	http://samtools.sourceforge.net
Deeptools v2.4.2	Ramírez et al., 2016	https://deeptools.readthedocs.io/en/develop/index.html
ngsPlot v2.61	Shen et al., 2014	https://github.com/shenlab-sinai/ngsplot/
R version 3.4.4	NA	https://www.r-project.org/

### Contact for Reagent and Resource Sharing

Further information and requests for resources and reagents should be directed to and will be fulfilled by the Lead Contact, Elmar Wolf (elmar.wolf@biozentrum.uni-wuerzburg.de).

### Experimental Model and Subject Details

#### Cell culture

Murine T-lymphoma ^MYC-Tet-Off^ cells (female) were grown in RPMI medium (Thermo Fisher Scientific) supplemented with 10% FBS (Sigma), 1% penicillin/streptomycin solution (Sigma), 1% glutamine solution (Thermo Fisher Scientific), 1% MEM non-essential amino acids (Thermo Fisher Scientific), and 50 μM β-mercaptoethanol (Sigma). Human U2OS (female), U2OS ^MYC-Tet-On^, HEK293 (female) and HeLa (female) cells, as well as murine NIH 3T3 (not applicable) cells, were cultured in DMEM (Thermo Fisher Scientific) supplemented with 10% FBS (Sigma) and 1% penicillin/streptomycin solution (Sigma). Human mammary epithelial cells (HMLE, female) were cultivated in advanced DMEM/F12 (Thermo Fisher Scientific) supplemented with 20 ng/ml EGF (human) (Thermo Fisher Scientific), 0.5 μg/ml Hydrocortisone (Sigma), 10 μg/ml Insulin (human) (Sigma), 1% penicillin/streptomycin solution (Sigma), 1% glutamine solution (Thermo Fisher Scientific) and 15 mM HEPES (pH 7.2). Human Neuroblastoma SH-EP cells (female) were verified by STR profiling and grown in RPMI medium (Thermo Fisher Scientific) supplemented with 10% FBS and 1% penicillin/streptomycin solution. All cell lines were cultured at 37°C, 5% CO_2_ and routinely screened and found negative for mycoplasma contamination in a PCR-based assay.

#### Cell line manipulation and generation

To generate stable cell lines for quantitative mass spectrometry, lentiviral packaging plasmids psPAX2 (Addgene #12260) and pMD2.G (Addgene #12259) were used. Lentivirus production was carried out in HEK293 cells and cell-free, virus-containing supernatant was used for infections. U2OS cells were infected and selected 48 h later, while T-lymphoma cells were infected twice every 24 h and selected 72 h after infection.

For plasmid transfection, polyethyleneimine (PEI; Sigma) reagent was used. For immunoprecipitation assays, transiently transfected HEK293 cells were harvested 24 h post transfection.

shRNAs against SPT5 were selected as described (Fellmann et al., 2013) and lentivirally transduced into the cell genome. For Figure S5F shRNA mirE1 against SPT5 was expressed, in Figures 5D and 5E a combination of shRNA mirE1 and mirE3 against SPT5 were induced by doxycycline for 48 h.

For siRNA transfections, the RNAiMAX transfection reagent (Thermo Fisher Scientific) was used with pools of siRNAs against MYC, CDK7, SPT5, TFIIE-α, TFIIE-β and negative control siRNA were purchased from Horizon Discovery Group. U2OS cell lines used for “MYC ON” and “MYC OFF” conditions were generated via transfection of siRNA against MYC mRNA on U2OS^MYC-Tet-On^ cells (Walz et al., 2014) followed by doxycycline (“MYC ON”) or ethanol (“MYC OFF”) addition for 12-15 h. To generate “MYC HIGH” cells, U2OS^MYC-Tet-On^ cells were transfected with non-targeting siRNA and then treated with doxycycline for 12-15 h. For ChIP sequencing cells were harvested 48 h after siRNA transfection and 12-15 h after addition of doxycycline, respectively. SH-EP cells were activated for NMYC by addition of 200 nM 4-hydroxytamoxifen (OHT) for 3 h.

Transcriptional inhibitor treatment was performed for 2.5 h with 100 μM DRB (5,6-dichlorobenzimidazone-1-β-D-ribofuranoside, Sigma) dissolved in DMSO. For proximity ligation assays and immunoprecipitations, inhibitor treatment was as follows: DRB (100 μM, 4 h), THZ1 (200 nM, 4 h), LDC067 (10 μM, 4 h), LDC4297 (0.5 μM, 4 h).

### Method Details

#### General cloning

All used primers are listed in Table S3. HA-RPB3 was ordered as gBlock from IDT and inserted into pRRL using AgeI and SpeI restriction sites (Primer #4). HA-MYC was cloned by PCR amplification using primers listed in Table S3 and inserted into pRRL using AgeI and SpeI restriction sites (Primer #1+3), for the N-terminal deletion of the first 143 amino acids Primer #2 was used. MYC in pCDNA3 was published earlier (Vo et al., 2016). N-FLAG SPT5 WT and mutants Δ1-Δ7 were cloned by PCR amplification using primers listed in Table S3 and inserted into pCMV3 using KpnI and NotI restriction sites (Primer #5-13). N-HA SPT5 WT and Δ7 mutant were cloned by PCR amplification using primers listed in Table S3 and inserted into pRRL using AgeI and SpeI restriction sites (Primer #14-16). GST-MYC^1163^ was cloned by PCR amplification using primers listed in Table S3 and inserted into pGex4T3 using EcoRI and XhoI restriction sites (Primer #17+18).

#### Mass Spectrometry

##### Complex purification for qMS

Native chromatin-associated Pol II complexes were isolated from T-lymphoma^MYC-Tet-Off^ cells following a published protocol (Aygün et al., 2008). Briefly, cells were treated with 1 μg/ml doxycycline (Sigma) for 16 h to deplete MYC or treated with ethanol. 600 million cells were harvested by centrifugation (300 g, 20 min, 4°C) and washed twice with ice cold PBS supplemented freshly with phosphatase and protease inhibitors (Sigma). Cytoplasmic lysis was carried out in extraction buffer I (10 mM HEPES pH 7.9, 0.34 M sucrose, 3 mM CaCl_2_, 2 mM magnesium acetate, 0.1 mM EDTA, 0.5% NP-40), and incubated for 20 min at 4°C with rotation. Nuclei were collected (3900 g, 20 min, 4°C) and washed once with extraction buffer I without NP-40. Nuclear lysis was carried out in extraction buffer II (20 mM HEPES pH 7.9, 3 mM EDTA, 10% glycerol, 150 mM potassium acetate, 1.5 mM MgCl_2_) followed by 20 min incubation (4°C, rotation) and 30 min centrifugation (13000 rpm, 4°C).

Precipitated chromatin was treated with nuclease incubation buffer (150 mM HEPES pH 7.9, 1.5 mM MgCl_2_, 150 mM potassium acetate) and sheared by sonicating four times 10 s with 45 s pausing (20% output). Benzonase (100 units/ml; Novagen) was added and the sample was incubated for 40 min at 4°C. Unsolubilized chromatin was pelleted by centrifugation (18,000 rpm, 30 min, 4°C). The soluble protein fraction was used in IP with 80 μl HA-coupled magnetic beads (Pierce Thermo Fisher Scientific) and an additional 200 units benzonase for 3 h at 4°C with rotation. Beads were washed thrice for 5 min each at 4°C with rotation in IP washing buffer (20 mM HEPES pH 7.9, 150 mM KCl, 0.5 mM EDTA, 10% glycerol) containing 0.1% Triton X-100, and then washed twice in buffer without Triton X-100. Protein complexes were eluted in 100 μl 1x LDS Sample Buffer (NuPAGE Thermo Fisher Scientific) by incubating for 30 min at 37°C. Then 1,4-dithiothreitol (DTT) was added to a final concentration of 50 mM and samples were heated at 95°C for 5 min.

For the isolation of native MYC complexes, 500 million T-lymphoma^MYC-Tet-Off^ and 200 million U2OS cells were harvested. For Figure 2A cells were lysed in low salt buffer (20 mM HEPES pH 7.8, 140 mM NaCl, 0.2 mM EDTA, 0.1% NP-40). Cells were lysed in extraction buffer (20 mM HEPES pH 7.9, 180 mM NaCl, 1.5 mM MgCl_2_, 10% glycerol, 0.2% NP-40) by douncing for 15 and 10 times for T-lymphoma and U2OS cells, respectively. After sonication and benzonase treatment, unsolubilized chromatin was precipitated and samples were further processed as for Pol II-associated proteins.

Eluted samples were prepared for mass spectrometry by reduction with 50 mM DTT (70°C, 10 min) and alkylation with 120 mM iodacetamide at room temperature, 20 min. Protein was precipitated by adding four volumes of acetone and incubating at −20°C overnight. Protein was pelleted by centrifugation (16,000 g, 20 min, 4°C), washed three times with acetone, and digested by LysC protease in 0.5% sodium deoxycholate for 2 h at 30°C followed by the addition of trypsin at 37°C overnight (enzyme/protein 1/200). SDC extraction was performed with ethyl acetate and 0.5% trifluoroacetic acid. Peptides were dried under vacuum to remove remaining ethyl acetate, and desalted via 3 discs of C18 Empore SPE Disks (3M) in a 200 μl pipet tip. Elution was performed twice with 60% acetonitrile and 0.1% formic acid, and eluates were dried under vacuum and stored at −20°C. Peptides were dissolved in 2% acetonitrile, 0.1% formic acid prior to nanoLC-MS/MS.

NanoLC-MS/MS was performed on an Orbitrap Fusion mass spectrometer (Thermo Fisher Scientific) equipped with a PicoView Ion Source (New Objective) and coupled to an EASY-nLC 1000 liquid chromatograph (Thermo Fisher Scientific). Peptides were loaded on capillary columns (PicoFrit, 30 cm x 150 μm ID, New Objective) self-packed with ReproSil-Pur 120 C18-AQ, 1.9 μm (Dr. Maisch), and separated with a 120-min linear gradient from 3% to 30% acetonitrile in 0.1% formic acid at a flow rate of 500 nL/min.

MS and MS/MS scans were acquired in the Orbitrap analyzer at a resolution of 60,000 and 15,000, respectively. HCD fragmentation with 35% normalized collision energy was applied. A Top Speed data-dependent MS/MS method with a fixed cycle time of 3 s was used. Dynamic exclusion was applied with a repeat count of 1 and an exclusion duration of 120 s; singly charged precursors were excluded from selection. Minimum signal threshold for precursor selection was set to 50,000. Predictive AGC was used with AGC a target value of 5e5 for MS scans and 5e4 for MS/MS scans. EASY-IC was used for internal calibration.

##### Mass spectrometry data analysis

Raw MS data files were analyzed with MaxQuant version 1.5.3.12 (Cox and Mann, 2008). Database search was performed with Andromeda within MaxQuant. The search was performed against the UniProt human reference proteome database (download date: 2016-12). Additionally, a database containing common contaminants was used. The search was performed with tryptic cleavage specificity with two allowed miscleavages.

Protein identification was under control of the false-discovery rate (< 1% FDR on protein and peptide level). In addition to MaxQuant default settings (e.g., at least 1 razor/unique peptide for identification, 2 allowed miscleavages), the search was performed for the following modifications: protein N-terminal acetylation, Gln to pyro-Glu formation (N-term. Q) and oxidation (on Met). For protein quantitation, LFQ intensities were used. Proteins with less than two identified razor/unique peptides were dismissed. Missing LFQ intensities in control samples were imputed with values close to baseline if intensities in the corresponding IP samples were present. Data imputation was performed with values from a standard normal distribution with a mean of the 5% quantile of the combined LFQ intensities and a standard deviation of 0.1. Missing logFC values for individual samples were imputed via MICE package in R, and the p values were recalculated using linear method in limma package in R. For Pol II proteomic analysis, interacting proteins (n = 101) were defined by log_2_FC(HA/Ctr) > 2 and q-value < 0.1. For MYC proteomic analysis, interacting proteins (n = 88) were defined by log_2_FC(HA/Ctr) > 1 and q-value < 0.1.

#### Co-immunoprecipitation

Cells were harvested in immunoprecipitation buffer (20 mM HEPES pH 7.9, 200 mM NaCl, 0.5 mM EDTA, 10% glycerol, 0.2% NP-40) containing phosphatase and protease inhibitors (Sigma), and lysed for 30 min at 4°C. Lysate was cleared by two sequential centrifugation steps. Dynabeads (20 μl of Protein A/G beads, Thermo Fisher Scientific) were pre-incubated, overnight at 4°C with rotation, in the presence of 5 g/l BSA and 2 μg antibody against HA (Santa Cruz Biotechnology, Y-11 X, #sc-805 X), FLAG (Sigma M2, #F3165), MYC (Abcam Y69, #ab32072) or SPT5 (Santa Cruz Biotechnology, A-3 X, #sc-133097 X). For HA-IP, HA-coupled magnetic beads (Pierce Thermo Fisher Scientific) were used. Lysate was added to washed beads and incubated for 3-6 h at 4°C. Elution of dynabeads was performed by heating the beads in 20-50 μl 1.5x Laemmli sample buffer (15 mM Tris pH 6.8, 3% SDS, 0.015% bromophenol blue, 10% glycerol, 1.5 mM 1,4-Dithiothreitol) for 5 min at 95°C. Elution of HA-coupled beads was carried out in 20 μl 1x LDS sample buffer (NuPAGE Thermo Fisher Scientific) by incubating for 30 min at 37°C. Afterward 1,4-Dithiothreitol (DTT) was added to a final concentration of 50 mM and samples were heated to 95°C for 5 min. Samples were analyzed by immunoblotting.

For the disruption assay HA-immunoprecipitation was performed. Afterward beads were resuspended in CDK7 buffer (100 mM NaCl, 20 mM HEPES pH 7.4, 4% glycerol, 1 mM 1,4-Dithiothreitol, 3 mM MgCl_2_, 1 mM ATP), CDK7 complex (Mat1, Cyclin H, CDK7) (Boehning et al., 2018) was added and mixture was incubated for 1 h at 30°C. Beads were washed and eluted in 1x LDS buffer as described above and samples were analyzed by immunoblotting.

#### Immunoblotting

Cells were lysed in RIPA lysis buffer (50 mM HEPES pH 7.9, 140 mM NaCl, 1 mM EDTA, 1% Triton X-100, 0.1% SDS, 0.1% sodium deoxycholate) containing protease and phosphatase inhibitors (Sigma) and incubated for 20 min at 4°C with rotation. Lysate was cleared by centrifugation and protein concentration was determined using the BCA assay. In some experiments, cells were directly lysed in 1x Laemmli sample buffer, and 2.5 units of benzonase (Novagen) were added to digest DNA for 15 min at room temperature. The cell lysate (same number of cells or amount of protein) was separated by BisTris-PAGE and transferred to PVDF membranes (Millipore). Membranes were probed using antibodies against: total Pol II (Santa Cruz Biotechnology N-20 X, #sc-899 X; Santa Cruz Biotechnology A-10, #sc-17798), pS2-Pol II (Abcam, #ab24758), MYC (Abcam Y69, #ab32072), SPT5 (Abcam, #ab126592), SPT6 (Novus Biologicals, #NB100-2582), HA (Santa Cruz Biotechnology Y-11 X, #sc-805 X), FLAG (Sigma M2, #F3165), GST (Sigma, #GE27-4577-01), CDK7 (Santa Cruz Biotechnology MO-1, #sc-56284), RPB2 (Santa Cruz Biotechnology E-12, #sc-166803), TRRAP (Bethyl Laboratories, #A310-373A), SPT4 (Santa Cruz Biotechnology A-12 X, #sc-515238X), Vinculin (Sigma, #V9131), CDK2 (Cell Signaling Technology 78B2, #2546S). For visualization the LAS3000 or LAS4000 Mini (Fuji) or Odyssey CLx Imaging System (LI-COR Biosciences) were used. Quantification was performed using ImageJ (version 1.50h) (Schneider et al., 2012) and Image Studio Lite (LI-COR Biosciences, Version 5.2.5) software (from at least triplicate blots).

#### Proximity ligation assays

Cells were plated at a density of 20,000 - 35,000 cells per 10 mm^2^ coverslip and left to adhere overnight before fixing with 4% paraformaldehyde. Fixed cells were permeabilized with 0.3% Triton X-100, washed in PBS, and treated with blocking solution (10% goat serum, 2% BSA, 5% sucrose in PBS) for 45 min. Cells were incubated overnight at 4°C with primary antibodies in blocking solution and then used in *in situ* proximity ligation assays (PLAs; Duolink kits, Sigma). Briefly, cells were treated for 1 h at 37°C with plus and minus probes directed at rabbit and mouse antibodies, respectively. Then probes were ligated for 30 min at 37°C. Next, *in situ* PCR amplification was done with Alexa 568- or Alexa 488-conjugated oligonucleotides for 2.5 h at 37°C. Samples were stained with Hoechst 33452 (Thermo Fisher Scientific) and Alexa 488- or Alexa 568-conjugated phalloidin (Thermo Fisher Scientific), mounted on microscope slides using Fluoromount (Sigma), and imaged under a confocal microscope (Nikon Ti-Eclipse or Leica SP8) with a 60X objective.

#### Immunofluorescence

Cells were plated, fixed and permeabilized as described for PLAs. Cells were incubated overnight at 4°C with primary antibodies in blocking solution, washed in Tris-buffered saline plus 0.1% Tween-20, and incubated for 1 h with fluorescently labeled secondary antibodies at room temperature. Then cells were stained with Hoechst 33342 (Thermo Fisher Scientific), mounted on microscope slides using Fluoromount (Sigma), and imaged under a confocal microscope (Nikon Ti-Eclipse) with a 60X objective.

#### Chromatin IP with reference exogenous genome spike-in followed by deep sequencing (ChIP-RX)

For each ChIP-RX sequencing experiment, 50 million cells per immunoprecipitation condition were fixed with formaldehyde (final concentration, 1%) for 5-10 min at room temperature. Fixation was stopped by adding 125 mM glycine for 5 min at room temperature. Cells were harvested in ice-cold PBS containing protease and phosphatase inhibitors (Sigma). All further used buffers also contained protease and phosphatase inhibitors. As exogenous control (spike-in), murine T-lymphoma or NIH 3T3 cells were added at a 1:10 cell ratio during cell lysis. Cell lysis was carried out for 20 min in lysis buffer I (5 mM PIPES pH 8.0, 85 mM KCl, 0.5% NP-40) and nuclei were collected by centrifugation at 1500 rpm for 20 min at 4°C.

Crosslinked chromatin was prepared in lysis buffer II (10 mM Tris pH 7.5, 150 mM NaCl, 1 mM EDTA, 1% NP-40, 1% sodium deoxycholate, 0.1% SDS) and fragmented by sonication (total duration, 20 min with 10 s pulses and 45 s pausing) or by using the Covaris Focused Ultrasonicator M220 for 50 min per ml lysate. Fragment size of 150-300 bp was validated by agarose gel electrophoresis. Chromatin was centrifuged for 20 min at 14,000 rpm at 4°C before IP. For each IP reaction, 100 μl Dynabeads Protein A and Protein G (Thermo Fisher Scientific) were pre-incubated, overnight with rotation in the presence of 5 g/l BSA and 15 μg antibody against: total Pol II (Santa Cruz Biotechnology A-10, #sc-17798), pS2-Pol II (Abcam, #ab5095), or SPT5 (Santa Cruz Biotechnology D-3 X, #sc-133217 X). Chromatin was added to the beads, and IP was performed for 6 h at 4°C with rotation. Beads were washed three times each with washing buffer I (20 mM Tris pH 8.1, 150 mM NaCl, 2 mM EDTA, 1% Triton X-100, 0.1% SDS), washing buffer II (20 mM Tris pH 8.1, 500 mM NaCl, 2 mM EDTA, 1% Triton X-100, 0.1% SDS), washing buffer III (10 mM Tris pH 8.1, 250 mM LiCl, 1 mM EDTA, 1% NP-40, 1% sodium deoxycholate; including a 5 min incubation with rotation), and TE buffer (Thermo Fisher Scientific). Chromatin was eluted twice by incubating with 150 μl elution buffer (100 mM NaHCO_3_, 1% SDS) for 15 min with rotation. Input samples and eluted samples were de-crosslinked overnight, and protein and RNA were digested with proteinase K and RNase A, respectively. DNA was isolated by phenol-chloroform extraction and ethanol precipitation, and analyzed by qPCR using StepOnePlus Real-Time PCR System (Thermo Fisher Scientific) and SYBR Green Master Mix (Thermo Fisher Scientific) or sequencing on the Illumina Next-Seq500.

For ChIP-RX sequencing, DNA was quantified using the Quant-iT PicoGreen dsDNA assay (Thermo Fisher Scientific). DNA library preparation was done using the NEBNext ChIP-Seq Library Prep Master Mix Set for Illumina (New England Biolabs) following the vendor’s instructions. Quality of the library was assessed on the Fragment Analyzer (Advanced Analytical) using the NGS Fragment High Sensitivity Analysis Kit (1-6,000 bp; Advanced Analytical).

##### ChIP-RX data analysis

FASTQ generation and quality verification were performed as described for 4sU-DRB sequencing. Data from murine T-lymphoma cells were used for normalization as described FASTQ files were aligned using Bowtie 2.27.0 for hg19 (Langmead et al., 2009) and mm10. Normalization was done based on scaling factor for each ChIP-Rx dataset calculated as in (Orlando et al., 2014) with the exception that mouse genomic reads were calculated instead of *Drosophila* genomic reads.

##### Processivity score calculations

To calculate the processivity score, ChIP RX BAM files were converted to BED files, and *coveragebed* was used to calculate read densities falling in distinct genomic regions. The proximal region was defined as the region from TSS+500 bp to TSS+1.5 kb whereas the distal region was from TES-1 kb to TES+2 kb. The processivity ratio was defined as the log2 of the ratio of pseudo-count added reads in the distal and proximal regions.

#### BAM file conversion and visualization

Base calling was performed using Illumina’s FASTQ Generation software v1.0.0, and sequencing quality was tested using the FastQC script. The reads were mapped to hg19 using Bowtie v2.2.7 (Langmead et al., 2009) (-N 1) or Bowtie v1.2. Reads falling in rRNA gene clusters and exons were removed using Bedtools v2.26.0 (Quinlan, 2014), and then the files were normalized to the mapped reads of files under comparison. For stranded 4sU sequencing, the reads were mapped using -nofw and -norc options for plus and minus stranded reads, respectively.

Normalized BAM files were sorted chromosome-wise using SAMtools v1.3 and converted to bedGraphs using Bedtools. For display purposes, the bedGraphs were visualized using a Genome Browser.

#### Average density plots for NGS data

Average density plots were generated from strand-based normalized BAMs with NGStools (Shen et al., 2014) of the indicated gene group and replotted by averaging the sense reads over both strands; the same procedure was followed for antisense reads. In case of replicates that were normalized to different reads, the densities were re-normalized to same read density for all curves. A similar strategy was used to generate metagene plots. For alternate poly-A sites density plot, only *the* promoter proximal sites were considered as generated in (Derti et al., 2012).

#### Gene set enrichment analysis

Gene set enrichment analysis (GSEA) was performed with the C2 and C5 gene sets from the Molecular Signature Database (MSigDB). The number of permutations was set to 1,000 and significant gene sets related to MYC were selected. Ranked sum analysis was carried out in the same way as GSEA although logarithmic processivity and directionality scores were set as the ranks.

#### 2D density plots for NGS data

The normalized BAM files were converted to BED files. Read densities were calculated using *coveragebed* for RefSeq genes in Bedtools, and binned and summarized in 2D using *stat_summary_2d* parameter of ggplots in R version 3.4.4.

#### ChIP quantitative PCR

For ChIP validation by quantitative PCR (qPCR) equal amounts of DNA and SYBR Green Master Mix (Thermo Fisher Scientific) containing 1 mM of the corresponding qPCR primers (Primer qPCR NCL_f: TACTGGGCAGGCTCAGTCTT; Primer qPCR NCL_r: GAAGATCCCGGAGCACGTA; Primer qPCR NegReg_f: TTTTCTCACATTGCCCCTGT; Primer qPCR NegReg_r: TCAATGCTGTACCAGGCAAA) were mixed. QPCR was performed using StepOnePlus Real-Time PCR System (Thermo Fisher Scientific) and analyzed in technical triplicates.

#### Protein expression and purification

DSIF (SPT4, SPT5) was expressed and purified as described (Vos et al., 2018). CDK7 was expressed and purified in context of the TFIIH kinase module (CDK7, Cyclin H, and MAT1) as previously described (Boehning et al., 2018).

#### Pull-down assays of recombinant proteins

pGex4T3 plasmids (GST and GST-MYC^1-163^) were transformed into BL21 *E.coli* and preculture was incubated overnight. LB-media was inoculated until an OD600 of 0.5. Overexpression was induced with 100 μM IPTG for 6 h. Bacteria were pelleted and lysed in STE buffer (150 mM NaCl, 10 mM Tris/HCl pH 8, 1 mM EDTA, 0.5 mM TCEP, protease inhibitors (Sigma)). Lysate was sonicated for three times 1 min (1 s pulse on, 1 s pulse off) and centrifuged. Washed Sepharose beads were incubated with lysate for 1 h at 4°C. After coupling, beads were washed with STE buffer.

For *in vitro* pull-down, GST or GST-MYC^1-163^ coupled beads were washed with pull-down buffer (100 mM NaCl, 20 mM Na-HEPES pH 7.5, 4% glycerol, 3 mM MgCl_2_, 1 mM 1,4-Dithiothreitol, 300 ng/ml BSA) and incubated with DSIF overnight at 4°C on rotating wheel. After pull-down, beads were washed with pull-down buffer and NETN buffer (20 mM Tris pH 8, 100 mM NaCl, 1 mM EDTA, 0.5% NP-40). Pull-down was eluted from beads in 2x Laemmli sample buffer (20 mM Tris pH 6.8, 4% SDS, 0.02% bromophenol blue, 13.4% glycerol, 2 mM 1,4-Dithiothreitol) at 95°C for 5 min.

MYC or Luciferase were expressed with the T7 Quick Coupled Transcription/Translation System (Promega) according to the manual. Briefly, 60 μl of reticulocyte-lysate were mixed with 2 μg of plasmid DNA and 4 μl of ^35^S-methionine (Hartmann Analytic) and incubated for 90 min at 30°C. Isolated DSIF was immobilized with 1 μg anti-SPT5 antibody (Santa Cruz Biotechnology A-3 X, #sc-133097 X) to a mixture of 20 μl of protein-A/G magnetic beads (Thermo Fisher Scientific) and incubated with 30 μl ^35^S-labeled protein for 3 h at 4°C. After washing with IP-buffer, bound proteins were eluted, separated by SDS-PAGE and visualized by Instant Blue (Expedeon) and autoradiography.

#### 4sU-DRB sequencing

Following 2.5 h of DRB treatment, cells were washed with PBS and released from transcription block by adding culture medium for indicated times. Then cells were treated with 200 μM 4-thiouridine (4sU; Sigma) for 10 min before being harvested in QIAzol reagent (QIAGEN).

Total RNA was extracted using miRNAeasy kit (QIAGEN) with on-column DNAase digestion, tested for quality on a Bioanalyzer (Bio-Rad) or Fragment Analyzer (Advanced Analytical Technologies), and quantified on a Nanodrop spectrophotometer. RNA (50-120 μg) was biotinylated using EZ-Link Biotin-HPDP (Pierce) in 0.2 mg/ml dimethylformamide in biotin labeling buffer (10 mM Tris pH 7.4, 1 mM EDTA) for 2 h with rotation at 25°C. Biotinylated RNA was cleaned by chloroform-isoamylalcohol (24:1) extraction in MaXtract (high density) tubes (QIAGEN). The aqueous phase was collected to which 1:10 volume of 5 M NaCl and an equal volume of isopropanol was added along with 1 μl of Glycoblue coprecipitant (Ambion). This was allowed to incubate at room temperature for 5 min followed by centrifugation at 20,000 g for 20 min at 4°C. Supernatant was discarded and resulting pellet was washed twice with 75% ethanol and centrifuging at 20,000 g for 10 min at 4°C, left to dry, and then dissolved in 100 μl RNase-free water.

Biotinylated RNA (100 μl) was pulled down using 50 μl of Dynabeads MyOne Streptavidin T1 beads (Invitrogen) suspended in 100 μl wash buffer (2 M NaCl with 10 mM Tris pH 7.5, 1 mM EDTA and 0.1% Tween 20) for 15 min at 25°C with rotation. Beads were magnetically separated and repeatedly washed with wash buffer. Finally, 4sU-RNA was eluted with 100 μl of freshly prepared 100 mM DTT in nuclease free water, and cleaned using RNeasy MinElute Spin columns (QIAGEN). The 4sU-RNA was quantified with the RiboGreen Assay (Invitrogen) and used for cDNA library preparation. cDNA libraries were prepared using Ultra RNA Library Prep kits (New England Biolabs) and the NEBNext® rRNA Depletion Kit (Human/Mouse/Rat) (New England Biolabs) in 16-19 PCR cycles, depending on the 4sU-RNA input. PCR products were assessed for size distribution and concentration on a Bioanalyzer (Bio-Rad) or on the Fragment Analyzer (Advanced Analytical) using the NGS Fragment High Sensitivity Analysis Kit (1-6,000 bp; Advanced Analytical).

##### Transcription elongation rate calculation

Pol II velocity was calculated using the computational pipeline TERate (Zhang et al., 2016). Briefly, each intron was split into 300-bp adjacent bins, and the expressed signal of each 300-bp bin was quantified. The 300-bp intron bins within the transcription start site (TSS) proximal region were randomly selected for “expressed bins.” Another set of intron bins within the TES proximal region of very long genes (≥60 kb) was randomly selected for “background bins.” “Transcribed bin” was defined as the bin where the tag density of expressed bins was greater than that of the background bin. For each selected gene, the position of the last two continuous transcribed bins was defined as the Pol II transcription edge. The transcription elongation distance for a gene was calculated from its TSS to the Pol II transcription edge. Finally, Pol II velocity was determined by dividing the transcription elongation distance by the transcription block release time.

To eliminate non-specific signals in 4sU-DRB sequencing and enhance the accuracy of elongation rate calculation, only genes ≥ 30 kb were studied. In addition, for genes with multiple isoforms and different TSSs, we studied the isoform with the shortest distance between the TSS and the start peak (a high signal proximal to the TSS).

##### Heatmaps for 4sU-DRB sequencing

Heatmaps were sorted for gene length or Pol II elongation rates as measured by independent 4-min and 8-min release 4sUDRB experiments in U2OS cells (unperturbed for MYC levels), and only the genes for which the elongation rates could be measured in all experiments were considered (n = 2,163). Heatmap generation was carried out using Deeptools (Ramírez et al., 2016). BAM files were converted to bigwigs with a bin size of 50. *computeMatrix* command was used with “reference point” as TSS and bin size of 50.–sortRegions parameter was set to *keep* and the sorted reference file (sorted for gene length or speed) was used as the input for -R parameter. Finally, the resulting matrix was used for *plotHeatmap* command with same -zmin and -zmax values for the set of conditions under comparison. The resulting heatmaps were overlaid using ImageJ after lookup table (LUT) assignment and contrast-enhanced to aid visibility.

#### 4sU sequencing

Cells were grown as above for 4sU-DRB sequencing without DRB treatment and with the duration of 200 μM 4sU treatment for 15 min. Total RNA extraction, biotinylation, streptavidin-based pull down and cleanup were done as described for 4sU-DRB sequencing.

cDNA libraries were prepared using Ultra Directional RNA Library Prep kits (New England Biolabs) and NEBNext® rRNA Depletion Kit (Human/Mouse/Rat) (New England Biolabs) in 16-19 PCR cycles, depending on the 4sU-RNA input. PCR products were assessed for size distribution and concentration on a Bioanalyzer (Bio-Rad) or on the Fragment Analyzer (Advanced Analytical) using the NGS Fragment High Sensitivity Analysis Kit (1-6,000 bp; Advanced Analytical).

##### Directionality/processivity score calculations

To calculate the processivity score, stranded BAM files were converted to BED files, and *coveragebed* was used to calculate read densities falling in distinct genomic regions. The proximal region was defined as the region from TSS+1 kb to TSS+2 kb, whereas the distal region was from TSS+5 kb to TSS+7 kb. The processivity ratio was defined as the log2 of the ratio of pseudo-count added reads in the distal and proximal regions. Only genes longer than 8 kb were considered for this calculation.

For the directionality score, the same BED files as above were used. For sense reads *coveragebed* was used to calculate pseudo-counted read densities from TSS-TES, while for antisense reads the region from TSS to TSS-1 kb was used. Directionality ratio was defined as the log2 of the ratio of sense reads to antisense reads. Genes smaller than 300 bp were excluded from this analysis.

##### Nascent RNA-based gene regulation calculation

To express gene regulation (in terms of log_2_FC), stranded BAM files were used to calculate read counts per gene. For each replicate, these reads were normalized for gene length and sequencing depth. Reads in exonic regions were not considered. Expression filter was set based on reads per kilobase per million (RPKM) averaged over all samples. Fold change was calculated as log2 ratio of averaged reads from replicates, and significance was calculated with Benjamini-Höchberg algorithm.

##### Heatmaps for directionality and processivity scores

Directionality and processivity scores were converted into a 2D matrix and row-wise Z scores were calculated. These were visualized on a heatmap using the Heatmap.2 function of *ggplot* in R version 3.4.4.

#### Analysis of tumor gene expression profiles

Normalized (RMA for microarray, FPKM for HT-seq data) gene expression data including meta information were obtained from GEO or TCGA Research Network (https://cancergenome.nih.gov/). Tumor samples were compared based on stage or type, and gene set enrichment analysis with default parameters was done by analyzing a set of 300 genes with low directionality score in high MYC. For Kaplan-Meier survival curves, patients were stratified based on the mean expression of 300 genes with low directionality score in high MYC. A cut-off of 10% was used for medulloblastoma samples (n = 34 with low expression) and of 50% for neuroblastoma samples (n = 44); p values were calculated with a two-tailed log-rank test.

### Quantification and Statistical Analysis

#### General statistics

All boxplots are generated using default values of parameters in R 3.4.4. For Figure 7G, the statistical significance was determined by an empirical gene set-based permutation test using R 3.4.4 with the p values provided in figure. For Figure 7I, the statistical significance was determined by two tailed log rank test using R 3.4.4 package with the p values provided in figure. For Figures 2H, 4C, 4D, 4I, 6E, 7B, 7H, S3C, S3H, S4G, S4I, S5D, S5E, S6D, S7B, S7C, S7E, and S7F the statistical significance was determined by a two tailed unpaired Wilcoxon test using R 3.4.4 package with the p values provided in each figure. For Figure S4E the statistical significance was determined by two tailed unpaired Student’s t test using R 3.4.4 package with the p values provided in the figure. For Figures 6F and 6G one way ANOVA was performed for p value calculation using linear regression model fitting in R 3.4.4.

#### PLA image processing and quantification

For image processing, ImageJ 1.50h (Schneider et al., 2012) was used. Proximity pairs were identified by measuring dot intensity using the ObjectCounter3D plugin. Briefly, centers of intensity were detected, assigned the same LUT color for all images, and processed with a Gaussian filter to aid visibility. The intensity centers were then counted, merged with phalloidin-488 and DAPI-stained channels, and displayed after correction for brightness and contrast, which was kept constant throughout the set of images under comparison. The intensity center count was confirmed with the counts reported by ObjectCounter3D. Intensity counts were then summed and tested for significance.

#### Immunofluorescence image processing

For image processing, ImageJ 1.50h was used. Z stack images were compressed into a single maximum intensity plane and assigned LUTs. Before displaying, the images were corrected for brightness and contrast, values of which were kept constant throughout the set of images under comparison.

### Data and Software Availability

The ChIP, 4sU and 4sU-DRB sequencing data have been deposited at the GEO (Gene Expression Omnibus) database under the accession number GSE115365 & GSE113861.

## Supplemental Information

Document S1. Figures S1–S7 and Table S3

Table S1. Mass Spectrometry Results of Pol II Interactome Data, Related to Figure 1 HA-RPB3 was expressed in T-lymphoma^MYC-Tet-Off^ cells treated with doxycycline (MYC OFF, 4 replicates) or in untreated cells (MYC ON, 5 replicates); after HA-immunoprecipitation, co-precipitating proteins were identified by label-free quantitative mass spectrometry. For all identified proteins, enrichment (log_2_FC) was calculated by comparing the signal to a control immunoprecipitation from cells not expressing HA-RPB3. Significantly enriched proteins are listed in bold. In case a protein was identified in at least 4 individual samples, enrichment for the missing experiments was imputed by the MICE package and put in brackets in order to calculate significance.

Table S2. Mass Spectrometry Results of MYC Interactome Data, Related to Figure 2 HA-MYC was expressed in T-lymphoma^MYC-Tet-Off^ or U2OS cells; after HA-immunoprecipitation, co-precipitating proteins were identified by label-free quantitative mass spectrometry. For all identified proteins, enrichment (log_2_FC) was calculated by comparing the signal to a control immunoprecipitation from cells not expressing HA-MYC. Significantly enriched proteins are listed in bold.

Document S2. Article plus Supplemental Information
