# Supplementary material for: MYC Recruits SPT5 to RNA Polymerase II to Promote Processive Transcription Elongation
Source: Mol Cell. 2019 May 16;74(4):674–687.e11. doi: 10.1016/j.molcel.2019.02.031 (PMC6527870; doi:10.1016/j.molcel.2019.02.031)
Supplement: Document S1. Figures S1–S7 and Table S3 [file mmc1.pdf]

Molecular Cell, Volume *74*

Supplemental Information

MYC Recruits SPT5 to RNA Polymerase II to Promote Processive Transcription Elongation

Apoorva Baluapuri, Julia Hofstetter, Nevenka Dudvarski Stankovic, Theresa Endres, Pranjali Bhandare, Seychelle Monique Vos, Bikash Adhikari, Jessica Denise Schwarz, Ashwin Narain, Markus Vogt, Shuang-Yan Wang, Robert Düster, Lisa Anna Jung, Jens Thorsten Vanselow, Armin Wiegering, Matthias Geyer, Hans Michael Maric, Peter Gallant, Susanne Walz, Andreas Schlosser, Patrick Cramer, Martin Eilers, and Elmar Wolf

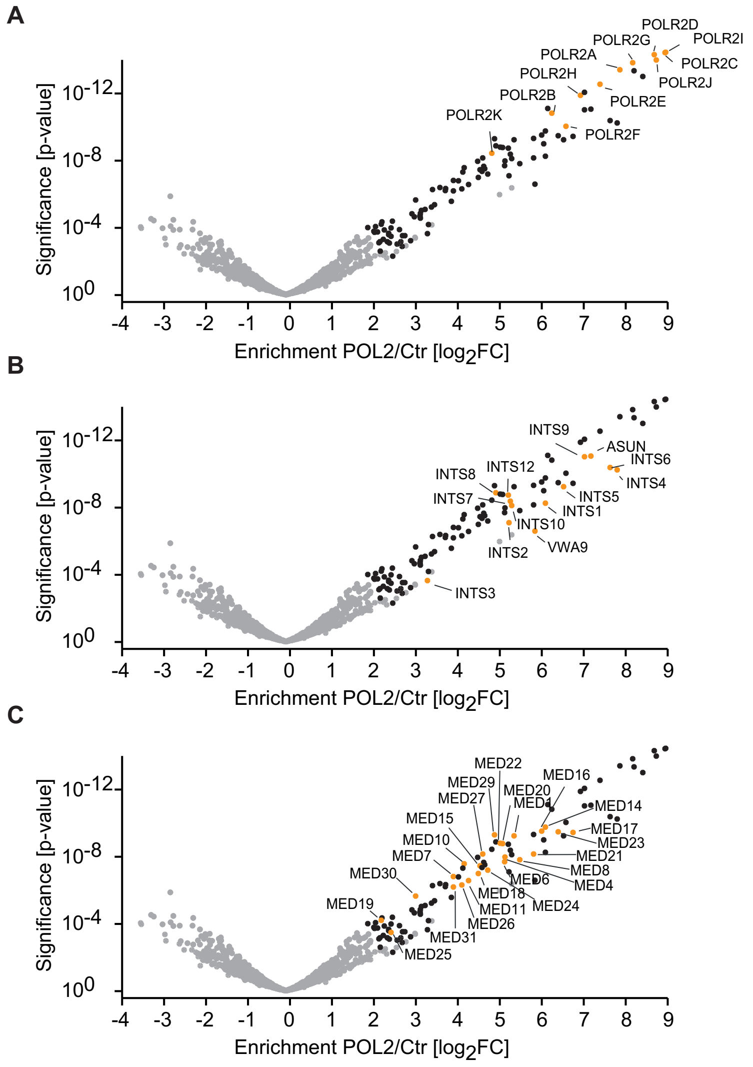

Figure S1. MYC mediates changes in POL2 complex composition, Related to Figure 1. Volcano plots of the POL2 interactome. **(A)** POL2 subunits. **(B)** Proteins of the integrator complex. **(C)** Proteins of the mediator complex. X-axes display the enrichment (log2FC) of proteins in HA-RPB3-expressing T-lymphomaMYC-Tet-Off cells compared with control cells not expressing HA-tagged protein (Ctr). Y-axes show the significance (p-value) of enrichment calculated from 5 biological replicate experiments. Interacting proteins were defined by log2FC(HA/Ctr) > 2 and q-value < 0.1; interacting proteins of interest are shown in orange and labeled. Values for all 101 interacting proteins are listed in Table S1.

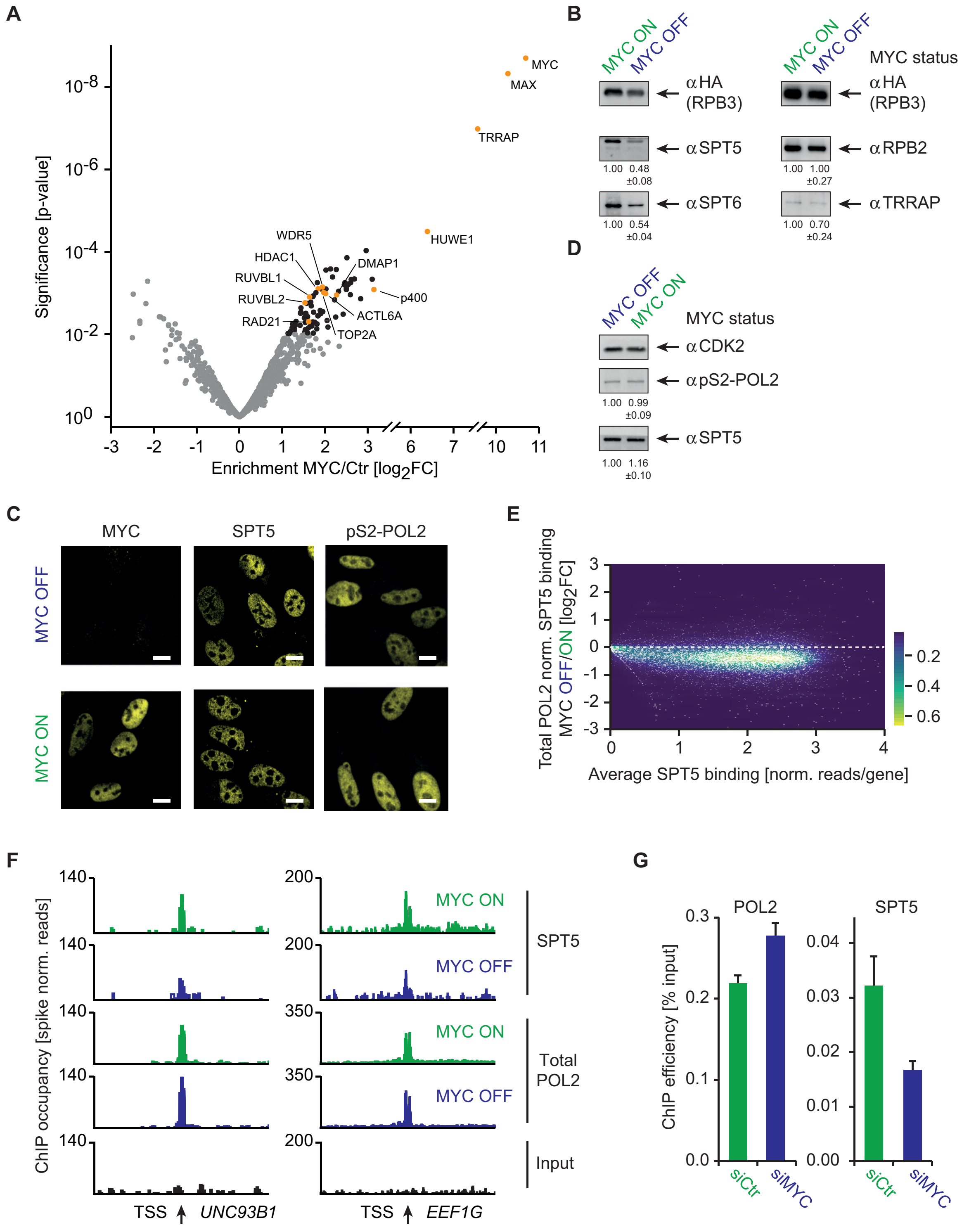

Figure S2. MYC-dependent recruitment of SPT5 to POL2 on chromatin, Related to Figure 2. (A) Volcano plot of the MYC interactome with characterized MYC-interacting proteins in orange. The X-axis displays the enrichment (log2FC) of proteins in HA-MYC-expressing cells compared to control cells not expressing HA-MYC. The Y-axis shows the significance (p-value) of enrichment calculated from 3 biological replicate experiments. Interacting proteins (n=88) were defined by log2FC(HA/Ctr) > 1 and q-value < 0.1. Values for all proteins are listed in Table S2. **(B)** Immunoblots of immunoprecipitation experiments in T-lymphomaMYC-Tet-Off cells. HA-RPB3 was immunoprecipitated in the absence and presence of MYC, and co-precipitated SPT5, SPT6, RPB2 and TRRAP were analyzed. Numbers below indicate the ratio of each protein to HA-RPB3 levels (MYC ON condition was set to 1). **(C)** Fluorescent images of U2OSMYC-Tet-On cells immunostained for MYC, SPT5 and pS2-POL2 in the absence or presence of MYC (scale bar: 5 µm). **(D)** Immunoblots of U2OSMYC-Tet-On cells showing pS2-POL2 and SPT5 levels in the absence and presence of MYC. CDK2 levels were analyzed as loading control. **(E)** Density plot demonstrating the drop in SPT5 associating with POL2 in the absence of MYC at the gene level. ChIP sequencing experiments of SPT5 and total POL2 were performed in the presence and absence of MYC in U2OS cells. To control for ChIP efficiency, ChIP-RX sequencing results were normalized to exogenous murine material (spiked-in control). The Y-axis displays the change in SPT5 binding between the absence and presence of MYC normalized to total POL2 binding (log2FC), and the X-axis depicts overall SPT5 binding normalized to gene length. Each white dot represents an individual gene with the color gradient scale representing density of reads overlaid on top. **(F)** Genome browser pictures of SPT5 and total POL2 from ChIP-RX sequencing experiments performed in the absence (blue) and presence (green) of MYC in U2OS cells. Reads were normalized to spiked-in controls. **(G)** qPCR results of ChIP experiments at the *NCL* gene. Chromatin bound by POL2 (left) or SPT5 (right) was enriched by specific antibodies in the presence (green) or absence (blue) of MYC and analyzed by qPCR in technical triplicates (represented as mean +/-SD; TSS, transcriptional start site; norm, normalized).

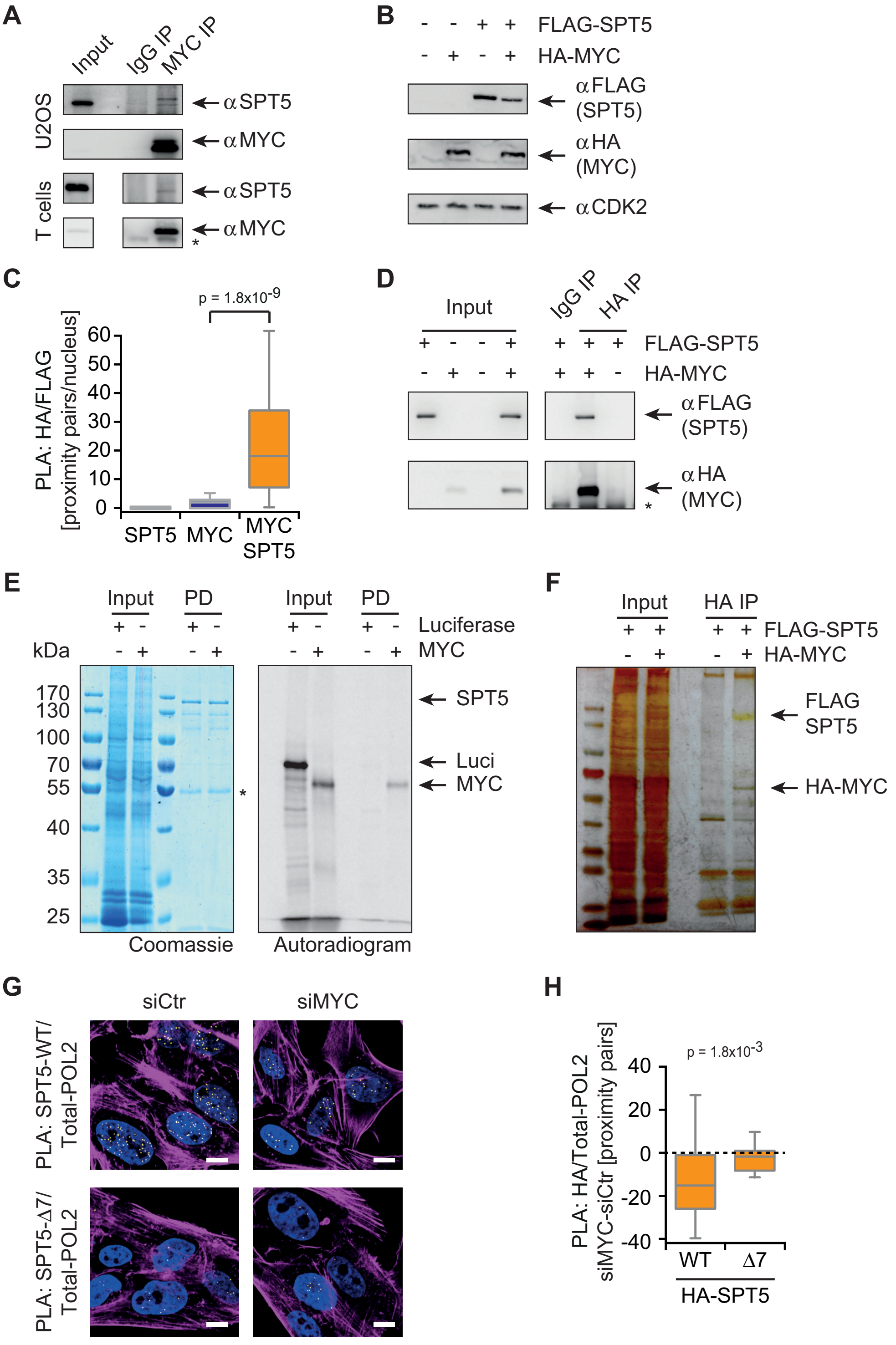

Figure S3. MYC recruits SPT5 by binding to its N-terminal region, Related to Figure 3. (A) Immunoblots of MYC immunoprecipitated from U2OS (top) or T-lymphoma cells (bottom). Co-precipitated endogenous SPT5 was analyzed by immunoblotting. Beads coupled to non-specific IgG were used as controls. * indicates the antibody heavy chain. **(B)** Immunoblot of HA-MYC and FLAG-SPT5 stably expressed in U2OS cells by lentiviral transduction. CDK2 was used as loading control. **(C)** Quantification of the PLAs shown in Figure 3B. **(D)** Immunoblots of immunoprecipitation experiments. FLAG-SPT5 and HA-MYC were overexpressed by transient transfection in HEK293 cells. HA-MYC was immunoprecipitated and co-precipitating FLAG-SPT5 was analyzed by immunoblotting. Cells not expressing HA-MYC or beads coupled to non-specific IgG were used as controls. * indicates the antibody heavy chain. **(E)** Polyacrylamide gel electrophoresis (PAGE) of proteins from pull-down assays visualized by Coomassie Brilliant Blue staining (left) and autoradiography (right). SPT5 was expressed together with SPT4 in *E. coli,* isolated and coupled to magnetic beads via SPT5-specific antibodies. Beads were then incubated with 35S -labeled MYC expressed *in vitro* or luciferase as controls. * indicates the antibody heavy chain. **(F)** Silver-stained PAGE gel. HA-MYC was immunoprecipitated from HEK293 cells expressing HA-MYC and FLAG-SPT5. Cells expressing only FLAG-SPT5 were used as control. Identity of the labeled bands (FLAG-SPT5 and HA-MYC) was confirmed by mass spectrometry. **(G, H)** Immunofluorescence images (G) and quantification (H) of PLAs between total POL2 and either an HA-tagged N-terminal deletion mutant (Δ7) or full-length (WT) SPT5 in the absence (siMYC) or presence (siCtr) of MYC in U2OS cells (Yellow dots: intensity centers of proximity pairs, blue: Hoechst stained nuclei, magenta: Phalloidin staining, scale bar: 5 μm). The boxplot shows the difference of proximity pairs between siCtr and siMYC treated cells.

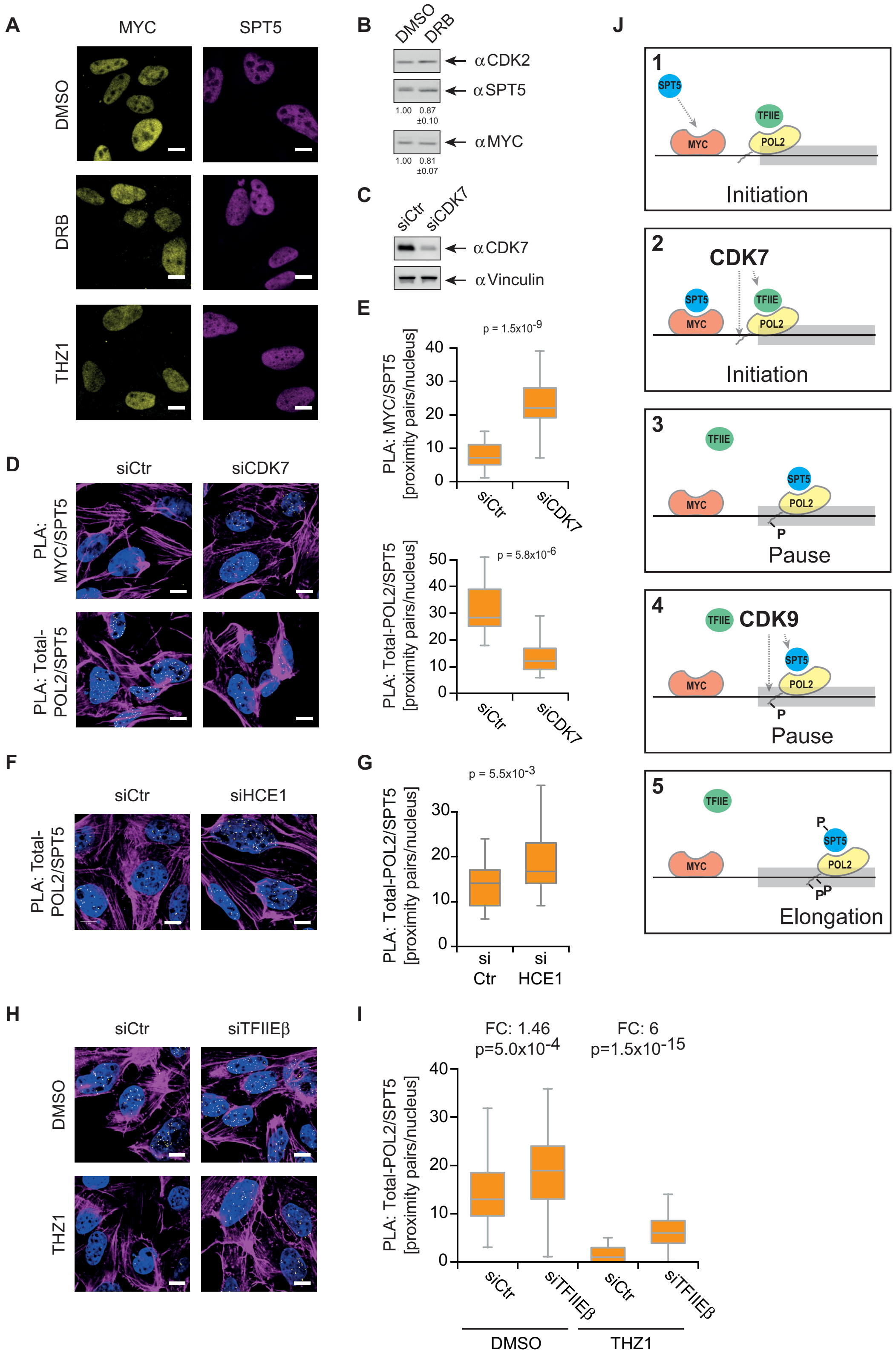

Figure S4. CDK7 activity is required for transfer of SPT5 from MYC to POL2, Related to Figure 4. (A) Immunofluorescence images of endogenous SPT5 and MYC after treatment of U2OS cells with transcription inhibitors (scale bars: 5 µm). **(B)** Immunoblot of MYC and SPT5 in U2OS cells treated with DRB (CDK2: loading control). **(C)** Immunoblot of CDK7 with or without siRNA depletion in U2OS cells (Vinculin: loading control). **(D)** Immunofluorescence images of PLAs between SPT5 and MYC (top) or between SPT5 and total POL2 (bottom) in the absence (siCDK7) or presence (siCtr) of CDK7 in U2OS cells (Yellow dots: intensity centers of proximity pairs, blue: Hoechst stained nuclei, magenta: Phalloidin staining, scale bar: 5 µm). **(E)** Quantification of PLAs between SPT5 and MYC (top) or SPT5 and total POL2 (bottom) upon CDK7 depletion as shown in (D). **(F, G)** Immunofluorescence images (F) and quantification (G) of PLAs. Proximity between total POL2 and SPT5 was analyzed in the absence (siHCE1) or presence (siCtr) of the human capping enzyme in U2OS cells (Yellow dots: intensity centers of proximity pairs, blue: Hoechst stained nuclei, magenta: Phalloidin staining, scale bar: 5 µm). **(H, I)** Immunofluorescence images (H) and quantification (I) of PLAs between SPT5 and total POL2 in U2OS cells depleted of TFIIEβ or control cells with or without THZ1 treatment. The number of proximity pairs upon inhibitor and depletion of TFIIE was quantified and is displayed by boxplots (yellow dots: intensity centers of proximity pairs, blue: Hoechst stained nuclei, magenta: Phalloidin staining, scale bar: 5 µm). **(J)** Model of MYC's effects on the transcriptional cycle of POL2. MYC recruits SPT5 to promoters. CDK7 converts POL2 receptive for SPT5 by promoting phosphorylation and dissociation of TFIIE and initiates transcription by CTD phosphorylation at Serine5 residues. Subsequently, CDK9 mediates pause release by phosphorylation of SPT5, NELF (not shown) and the Serine2 residues of the POL2-CTD.

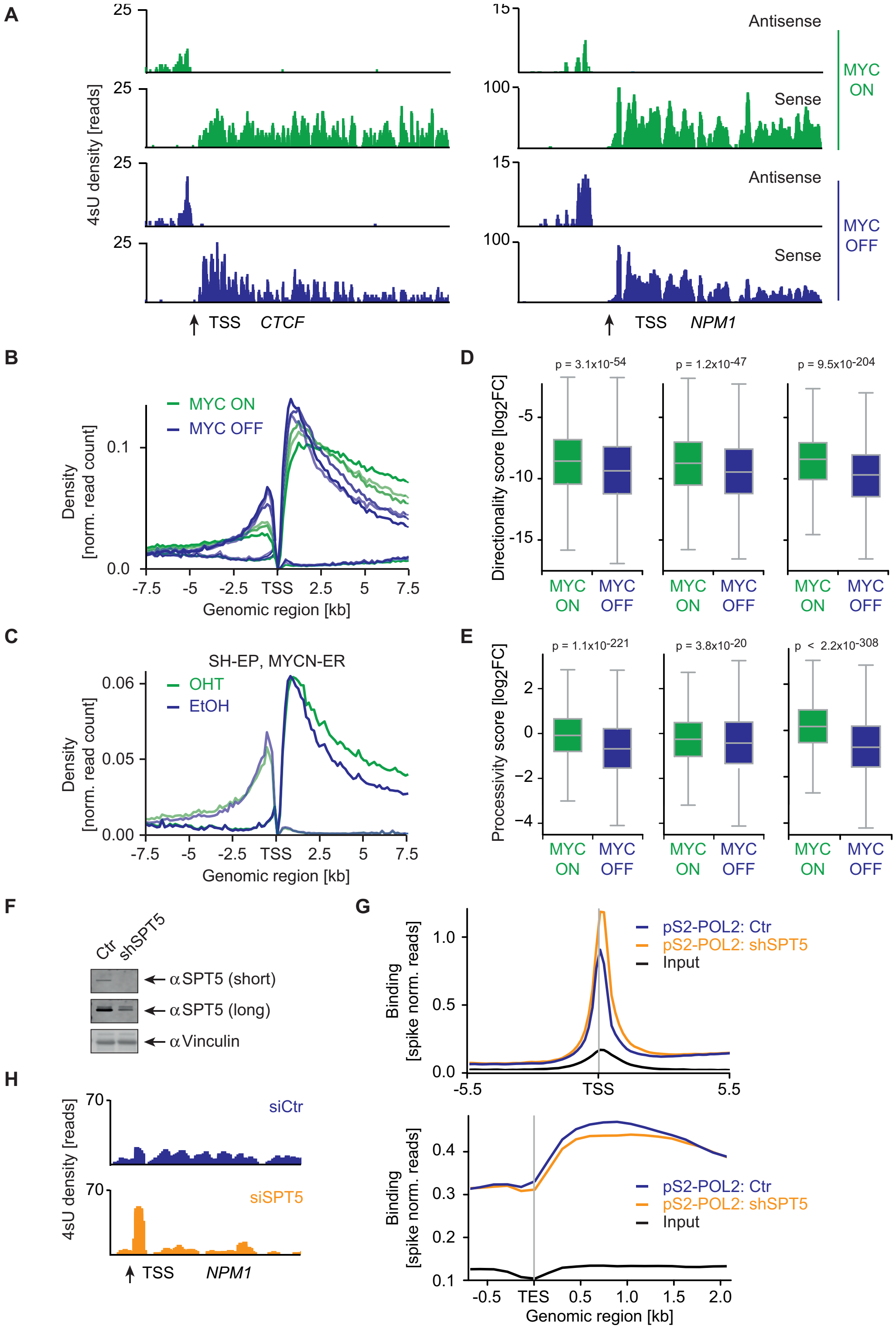

Figure S5. MYC-mediated transfer of SPT5 is required to maintain POL2 processivity, Related to Figure 5. (A) Genome browser picture of nascent RNA. Example of normalized directional 4sU signal at the *CTCF* and *NPM1* genes from U2OS cells in the absence and presence of MYC. **(B)** Average read density of 4sU sequencing experiments in U2OS cells in the absence and presence of MYC as in Figure 5C. All three replicates are depicted as individual curves showing spatial distribution of reads independently aligned to sense and antisense strands within 7.5 kb of the TSS. **(C)** Average read density of 4sU sequencing experiments in SH-EP cells from GSE113861. MYCN was activated by 4-hydroxytamoxifen (OHT) in cells expressing a MYCN-ER fusion protein. Curves show spatial distribution of reads independently aligned to sense and antisense strands within 7.5 kb of the TSS of all genes. **(D)** Boxplots of directionality scores for expressed genes in U2OS cells, calculated by dividing sense by antisense reads as shown in Figure 5A. All three replicates are shown. **(E)** Boxplots of processivity scores for expressed genes in U2OS cells, calculated by dividing distal by proximal reads as shown in Figure 5A. All three replicates are shown. **(F)** Immunoblot of U2OS control cells or depletion of SPT5 by an inducible, lentiviral construct encoding an shRNA. Depletion of SPT5 was induced by doxycycline (shSPT5) and expression of SPT5 was compared to control cells treated with ethanol (Ctr). SPT5 is shown with short and long exposure (Vinculin: loading control). **(G)** Average read density of POL2 ChIP sequencing experiments around transcriptional start site (TSS, top) and transcriptional end site (TES, bottom). ChIP-RX sequencing was done with antibodies precipitating pS2-POL2 in U2OS cells depleted for SPT5 (orange) and in control cells (blue) not expressing shSPT5. To control for ChIP efficiency, reads were normalized to exogenous murine material (TSS: transcriptional start site; norm.: normalized, black line: input). **(H)** Genome browser pictures of the *NPM1* gene. Normalized 4sU sequencing reads from U2OS cells visualizing transcriptional activity in U2OS cells upon siRNA-mediated depletion of SPT5 (orange) or control cells (blue).

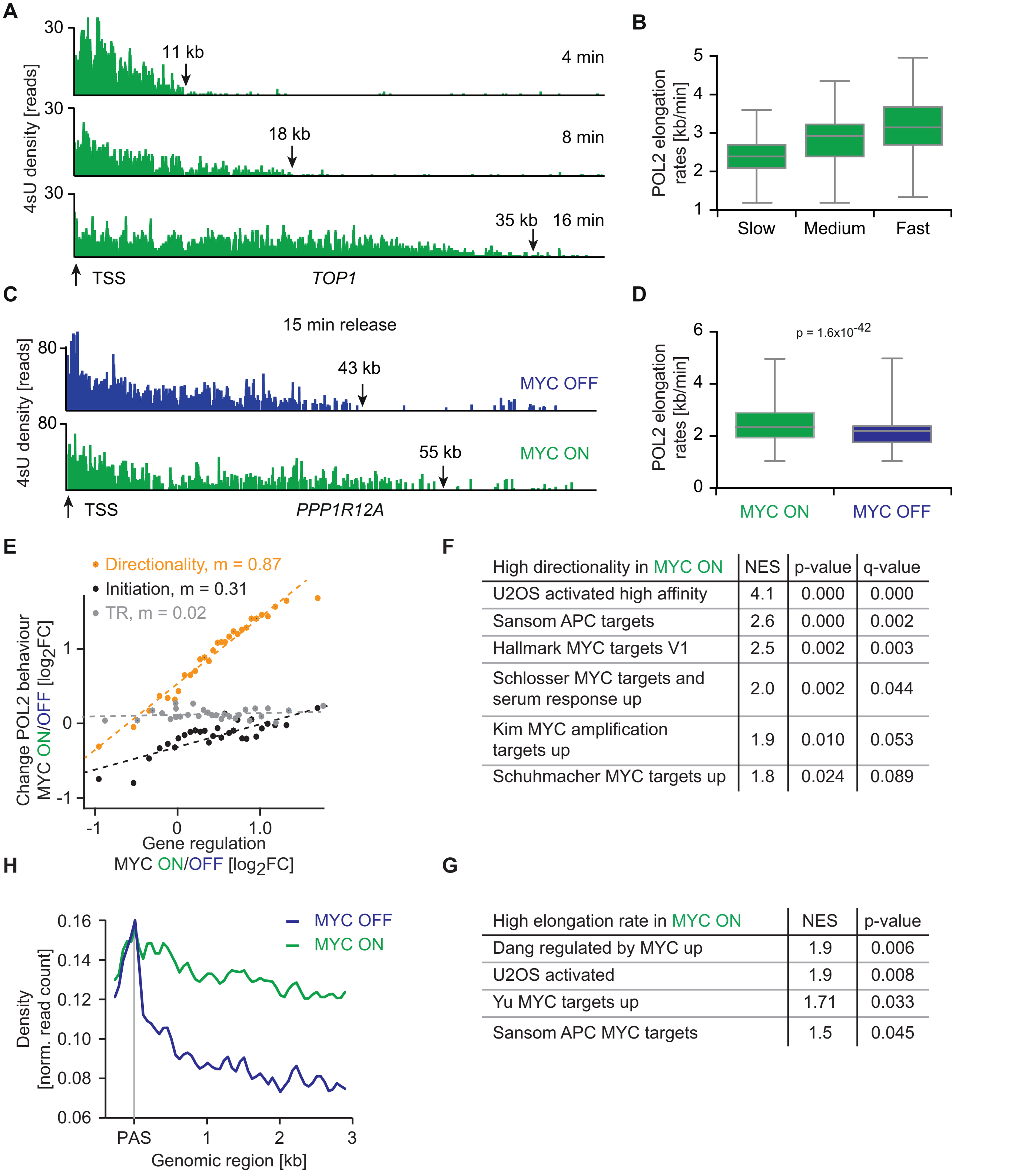

High directionality in MYC ON	NES	p-value	q-value
U2OS activated high affinity	4.1	0.000	0.000
Sansom APC targets	2.6	0.000	0.002
Hallmark MYC targets V1	2.5	0.002	0.003
Schlosser MYC targets and serum response up	2.0	0.002	0.044
Kim MYC amplification targets up	1.9	0.010	0.053
Schuhmacher MYC targets up	1.8	0.024	0.089

High elongation rate in MYC ON	NES	p-value
Dang regulated by MYC up	1.9	0.006
U2OS activated	1.9	0.008
Yu MYC targets up	1.71	0.033
Sansom APC MYC targets	1.5	0.045

Figure S6. MYC is required to globally maintain high transcription elongation rates, Related to Figure 6. (A) Genome browser pictures of nascent RNA showing the transcriptional wave front of POL2 at different times after release from DRB inhibition at the *TOP1* gene. **(B)** Elongation rates from 4sU-DRB sequencing 4 min after release from DRB inhibition. Genes were classified as “Slow”, “Medium” and “Fast” based on elongation rate measurements from an independent experiment (8 minutes after DRB release). **(C)** Genome browser pictures of the *PPP1R12A* gene. The wave front of the 4sU-DRB sequencing experiment indicates the location of POL2 15 min after release from DRB treatment in the absence and presence of MYC. **(D)** POL2 elongation rates in the absence and presence of MYC measured 15 min after DRB release. **(E)** Correlation between MYC-mediated changes in gene regulation and three parameters of POL2 behavior. Mean values of bins containing an equal number of genes are shown. The X-axis displays MYC-mediated changes in gene regulation (log2FC) in the presence and absence of MYC from 4sU sequencing experiments. The Y-axis depicts MYC-mediated changes in directionality (orange), 4sU read density in the promoter region (“initiation”, black), and POL2 traveling ratio (TR) based on ChIP sequencing of total POL2 (grey). m indicates slope of regression curve. **(F, G)** Gene sets most affected by MYC-mediated effects on elongation. Gene set enrichment analysis was performed on genes ranked for change in directionality score (F) and elongation rate (G) between cells with and without MYC expression. A positive normalized enrichment score (NES) indicates gene sets with bi-directional transcription (F) or reduced elongation rate (G) upon depletion of MYC. **(H)** Average read density of 4sU sequencing experiments in U2OS cells in the absence (blue) and presence (green) of MYC. Reads are centered at the first alternative polyadenylation-site (PAS) downstream the promoter.

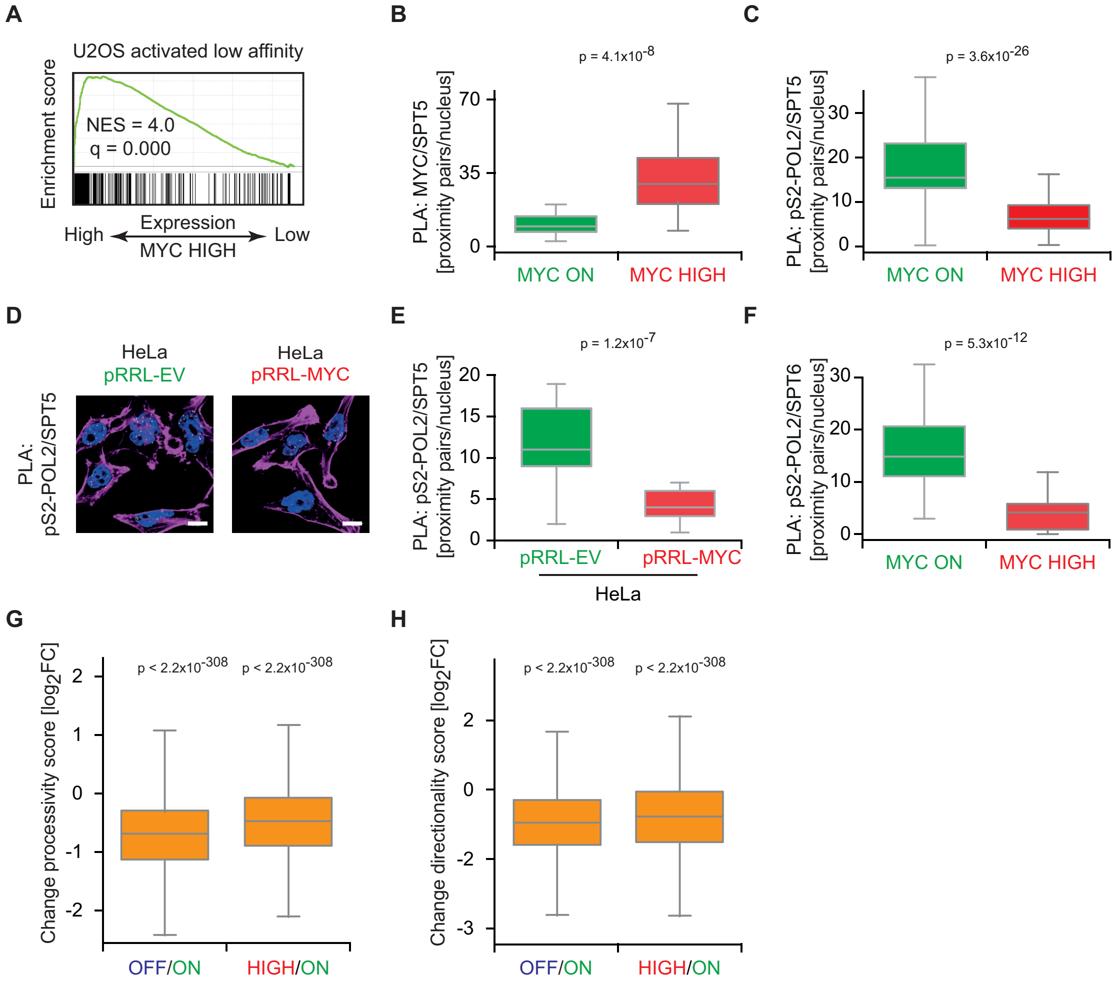

I

Experimental MYC signatures	NES	p-value	q-value
Kim MYCN amplification targets down	3.4	0.000	0.000
Dang regulated by MYC down	2.5	0.000	0.002
Kim MYC amplification targets down	2.0	0.006	0.030

MYC repressed functional processes	NES	p-value	q-value
KEGG TGFβ signalling pathways	3.0	0.000	0.000
GO Regulation of immune response	2.9	0.000	0.001
Schoen NFκB signaling	2.8	0.000	0.000
Biocarta TGFβ pathway	2.5	0.000	0.002
Peng Glutamine deprivation up	2.4	0.000	0.004
KEGG Focal adhesion	2.3	0.000	0.005

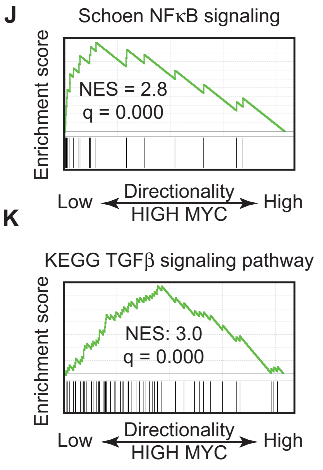

Figure S7. Oncogenic MYC levels sequester SPT5 away from POL2, Related to Figure 7. (A) Plot of gene set enrichment analysis performed with 4sU sequencing data shows the normalized enrichment score (NES) and q-value for the “U2OS activated low affinity” gene set (Lorenzin et al., 2016) regulated in U2OSMYC-Tet-On cells upon induction of MYC (high vs. medium). Vertical black bars indicate the position of low affinity MYC-activated target genes in the 4sU dataset, and the enrichment score is shown as a green line. This analysis demonstrates that genes induced at high MYC levels are similar irrespectively if measured at the nascent (4sU sequencing) or mature (RNA sequencing) level. **(B, C)** Quantification of the PLAs shown in Figure 7C, D. **(D, E)** Immunofluorescence images (D) and quantification (E) of PLAs between pS2-POL2 and SPT5 in HeLa cells overexpressing MYC (pRRL-MYC) or control HeLa (EV: empty vector) cells transduced with lentiviral expression constructs (Yellow dots: intensity centers of proximity pairs, blue: Hoechst stained nuclei, magenta: Phalloidin staining, scale bar: 5 µm). **(F)** Quantification of the PLAs shown in Figure 7F. **(G)** MYC-mediated effects on processivity, calculated for U2OS cells expressing low (OFF), physiological (ON) or oncogenic (HIGH) levels of MYC, from read densities of 4sU sequencing experiments. The effect of MYC was visualized by determining the MYC-mediated change in processivity (log2FC) for every gene. Negative values demonstrate an increase in processivity at physiological levels of MYC (OFF/ON) or a decrease of processivity at oncogenic levels (HIGH/ON). **(H)** MYC-mediated effects on promoter directionality. Directionality scores were calculated for U2OS cells expressing low (OFF), physiological (ON) or oncogenic (HIGH) levels of MYC, from read densities of 4sU sequencing experiments. The effect of MYC was visualized by determining the MYC-mediated change in directionality (log2FC) for every gene. **(I, J, K)** Gene set enrichment analysis with genes ranked by change in directionality score between normal and high (oncogenic) levels of MYC. (I) Gene sets with the highest normalized enrichment scores (NES). (J, K) Enrichment plots for the “Schoen NFκB” gene set (J) and “KEGG TGFβ signaling pathway” gene set. Vertical black bars indicate the position of genes in the ranked gene list; the enrichment score is shown as a green line. This analysis shows that classic MYC-repressed genes and tumor-suppressive genes (e.g. immune response genes) have the lowest directionality at high MYC levels.

Table S3. List of primers used for cloning. Related to STAR Methods. Table depicts primers used for cloning of HA-RPB3, HA-MYC, FLAG-SPT5, HA-SPT5, SPT5-truncated mutants (HA- or FLAG-tag), MYC-Mutant AA144-439 (HA-tagged) and GST-$MYC^{1\text{-}163}$. Corresponding vector backbone is put in brackets.

#	Name	Sequence
1	MYC-WT-f (pRRL)	GTACCGGTATGCCCCTCAACGTTAGCTT
2	C-HA-MYC-r (pRRL)	GGACTAGTTTAGGCGTAATCTGGAACATCG
3	MYC-(144-439)-f (pRRL)	GTACCGGTATGCTCGTCTCAGAGAAGCTGGC
4	C-HA-RPB3-f (pRRL)	GTACCGGTATGCCGTACGCCAACC
5	N-FLAG-SPT5-f (pCMV3)	CGGGGTACCATGGATTACAAGGAT
6	SPT5_Δ1_r (pCMV3)	ATAGTTTAGCGGCCGCTCAGGCCACCTCCTTCAC
7	SPT5_Δ2_r (pCMV3)	ATAGTTTAGCGGCCGCTCACACCTTCAGCTCATG
8	SPT5_Δ3_r (pCMV3)	ATAGTTTAGCGGCCGCTCAACGGTCCACAGAGAT
9	N-FLAG-SPT5_Δ4_f (pCMV3)	CGGGGTACCATGGATTACAAGGATGACGACGATAAGGGTG GAGGCGGTAGCGAGTATGCTTTCGATGATGA
10	N-FLAG-SPT5_Δ5_f (pCMV3)	CGGGGTACCATGGATTACAAGGATGACGACGATAAGGGTG GAGGCGGTAGCCGGCTCACCACGGTGGGCTC
11	SPT5-r (pCMV3)	ATAGTTTAGCGGCCGCTCAGGCTTCCAGGAGCTTCC
12	N-FLAG-SPT5_Δ6_f (pCMV3)	CGGGGTACCATGGATTACAAGGATGACGACGATAAGGGTG GAGGCGGTAGCATGAGCAGGGGCCGGGGCCG
13	N-FLAG -SPT5_Δ7_f (pCMV3)	CGGGGTACCATGGATTACAAGGATGACGACGATAAGGGTG GAGGCGGTAGCGTGCTCAAAGTGGTGAAGGA
14	N-HA-SPT5-f (pRRL)	GTACCGGTATGTACCCATACGATGTTCCAGATTACGCCAGC TCGGACAGCGAG
15	SPT5-r (pRRL)	GGACTAGTTCAGGCTTCCAGGAGCTTC
16	N-HA-SPT5_Δ7-f (pRRL)	GTACCGGTATGTACCCATACGATGTTCCAGATTACGCCAAC CTGAAACCAAAGTCCTG
17	MYC-f (pGex4T3)	CCCGAATTCGCCCCTCAACGTTAGCTTC
18	MYC-r (pGex4T3)	GGGCTCGAGTCAGTTCGGGCTGCCGCTGTCT
